# Relationships of post-stroke fatigue with mobility, recovery, performance, and participation-related outcomes: a systematic review and meta-analysis

**DOI:** 10.3389/fneur.2024.1420443

**Published:** 2024-10-08

**Authors:** Jibrin S. Usman, Thomson W. L. Wong, Shamay S. M. Ng

**Affiliations:** Department of Rehabilitation Sciences, The Hong Kong Polytechnic University, Kowloon, Hong Kong SAR, China

**Keywords:** stroke, post-stroke fatigue, mobility, recovery, performance, walking, motor function, participation

## Abstract

**Background:**

Effective post-stroke mobility, recovery, performance, and participation are key goals for stroke survivors. However, these outcomes may be hindered by post-stroke fatigue (PSF), which can affect numerous aspects of post-stroke mobility, recovery, performance, functioning, community participation, and return to work. This review aimed to assess the scientific evidence on the relationship between PSF and mobility function, functional recovery, functional performance, and participation-related outcomes among stroke survivors.

**Method:**

A comprehensive search of Cochrane Central, PubMed, Embase, and Web of Science (WoS) databases was conducted from inception to December 2023. Observational, cross-sectional, and longitudinal studies were included. The methodological quality of the included studies was assessed using the National Institute of Health’s quality assessment tool, while the risk of bias was assessed using the Quality in Prognostic Studies tool. A total of 28 studies (n = 2,495 participants, 1,626 men, mean age ranging from 52.5 ± 9.5 to 71.1 ± 9.9 years) were included. The data analysis was conducted using narrative and quantitative synthesis. Fixed and random effects meta-analyses were conducted to explore the relationships between PSF and relevant outcomes.

**Results:**

Chronic PSF was found to have significant negative correlations with mobility (meta r = −0.106, *p* < 0.001), balance performance (meta r = −0.172; 95%; *p* = 0.004), and quality of life (meta r = −0.647; *p* < 0.001). It also showed significant positive correlations with stroke impairment (meta r = 0.144, *p* < 0.001) and disability (meta r = 0.480, *p* < 0.001). Additionally, exertion/acute PSF had significantly negative correlations with walking economy (meta r = −0.627, *p* < 0.001) and walking endurance (meta r = −0.421, *p* = 0.022). The certainty of evidence was deemed moderate for these relationships.

**Conclusion:**

Our findings indicate that higher levels of PSF are associated with poorer mobility, balance, and participation, as well as greater disability and stroke impairment. Future studies, especially prospective longitudinal and randomized controlled trials, are warranted to substantiate our findings.

**Systematic review registration:**

PROSPERO, identifier: CRD42023492045.

## Introduction

Stroke survivors experience a range of impairments and functional limitations that manifest in various combinations (1). In addition to this, they may experience various post-stroke symptoms, such as fatigue, pain, and spasticity, which often occur concurrently and can significantly influence mobility, motor function, physical function, and activities of daily living (ADLs), adding to the overall burden and hindering recovery (2). Fatigue in stroke survivors can be classified as either chronic or acute (exertion related), with two distinct characteristics (3). Exertion/state fatigue is characterized by its immediate onset and recovery time (3), while chronic fatigue is caused by long periods of accumulation of acute fatigue (4) or the gradual progression of mental fatigue, potentially triggered by daily tasks (4, 5). Post-stroke exertion fatigue is typically experienced after intense physical or mental exertion (5). Additionally, fatigue can be categorized as mental or peripheral (physical), with underlying mechanisms often associated with autonomic diseases (4).

PSF is associated with feelings of mental, physical, and overall exhaustion, with a variation in fatigue levels and activity (6). Its characteristics vary from general fatigue to a certain degree, and it can occur without any specific exertion (7). PSF is a common post-stroke deficit typified by complex multifactorial phenomena (8) and is a frequent, incapacitating health issue due to the complex interactions of numerous factors (9). Fatigue and sleepiness commonly exist together due to lack of sleep and are usually combined under the concept of tiredness by patients. However, they are two separate but interrelated terms (10). The two terms fatigue and sleepy are different, with the suggestion that clinicians and researchers should be cautious when using these terms interchangeably (11). Fatigue is an overwhelming feeling of tiredness, lacking energy, and a sense of exhaustion related to diminished physical and/or cognitive performance, whereas sleepiness is a pervasive phenomenon felt not just as a symptom in various disorders but as a normal state of physiology in most persons during any given 24-h duration (10). Additionally, disorder is inferred both when sleepiness becomes pervasively present or when it is absent, and abnormality is considered when it does not happen when needed or happens at unsuitable periods (10).

Post-stroke fatigue (PSF) is prevalent in stroke survivors (12–15) and affects their daily functioning, and quality of life is a commonly disregarded problem (3). It affects participation, emotions, cognitive performance, and ADLs and can diminish the ability to carry out the expected ADLs (6). PSF, which can have numerous adverse consequences (16), is significantly associated with functional impairments, disability, and diminished quality of life among stroke survivors in several areas (1, 14). It has also been shown to have a negative influence on the survivors’ cardiopulmonary function (13), and fatigue hinders their community integration (12). Thus, fatigue is a major symptom that affects numerous physical functions, such as ADLs and mobility, in stroke survivors.

The effects of fatigue can be lifelong (17, 18) and may impact post-stroke functional recovery and outcomes (2, 18). Moreover, PSF is associated with dual-task performance (19), both cognitive and motor performances (8), lower extremity mobility (20), and lower limb motor tasks, including balance and gait, with more challenges to navigating in complex settings likely to be observed in those with higher fatigue (8). Other studies have found that fatigue is weakly related to post-stroke gait performance (19), inextricably related to affective disorders (21), and also related to poor functional outcomes in young stroke survivors (18).

The summary above shows that most of the studies reporting the relationships or associations between PSF and variables of interest have not expounded the strength, direction, or extent of the relationships. This precludes the drawing of definite conclusions regarding the relationships. Therefore, there is a need to synthesize the scientific evidence of how and to what extent PSF influences the mobility, recovery, performance, and participation of stroke survivors. Such evidence will guide stroke rehabilitation professionals in formulating better rehabilitation strategies by targeting PSF, mobility, recovery, functioning, and participation. Despite the known importance of these outcomes in stroke survivors, to the best of our knowledge, this will be the first systematic review and meta-analysis that determine the scientific evidence on how and to what extent PSF influences these outcomes in stroke survivors.

## Methods

This review underwent PROSPERO registration (registration number: CRD42023492045), and the Preferred Reporting Items for Systematic Reviews and Meta-Analyses (PRISMA) guideline was applied for conducting the review.

### Search strategy

The EMBASE, PubMed, PEDro, Cochrane Central Library, and Web of Science online databases have been searched from inception till December 2023. The PICOS approach was applied to form the search terms for searching the databases. All of the databases have been searched in compliance with their unique requirements, and there was a truncation of search terms and utilization of medical subject headings (MeSH) terms where necessary and appropriate. The search process adopted in the databases is in Supplementary material S1. In addition, the reference lists of the retrieved studies and reviews were searched manually. Relevant articles obtained from the general literature search were also involved in the review. The results of the searches were exported to Endnote, duplicate studies were discarded, and the remaining articles were subjected to title and abstract screening, followed by a full-text screening process. The search was conducted independently by a researcher (JSU), and the results were confirmed by two other researchers (TWLW and SSMN).

### Eligibility selection criteria

Studies that met the following inclusion criteria were included in the review: studies that (i) included adult stroke survivors; (ii) were published in English; (iii) reported the relationship between PSF and any of the outcomes of interest, namely related to mobility, functional recovery, functional performance and participation, including but not limited to gait, balance, falls, ADLs, quality of life (QOL), international classification of functioning (ICF) domains, self-confidence, motor function and muscular strength; (iv) were cross-sectional (CRS), longitudinal, cohort, observational, prospective or correlational with full texts available. Conference abstracts, review articles, dissertations/theses, and commentaries have been excluded.

### Study selection and data extraction

Initially, upon removal of studies that were duplicated, the titles and abstracts of the remaining studies were screened independently by two researchers (JSU and TWLW) against the eligibility criteria. Any differences between the two researchers concerning the involvement of a study were resolved by contacting a third researcher (SSMN) to resolve the differences. During the full-text screening, the full texts of the appropriate studies were accessed, and the data as follows were taken out: study author(s), year of publication and location of study, study objective, study design, sample size, characteristics of the patient population, outcome measures, outcomes assessed, and findings/conclusions. All the extracted data were documented in a Microsoft Excel file. The extracted data and the characteristics of the included studies are presented in Table 1.

**Table 1:** Included study characteristics.

Study author, year Location	Study objective	Study design	Sample size	Characteristics of the patient population	Assessment scale (s) type for the exposure	Outcome measure(s)	Outcome(s)	Findings/conclusion
Ali et al., 2022 Egypt	To determine the post-stroke fatigue (PSF) prevalence in chronic stroke patients and to investigate its correlation with their functional recovery.	Cross-sectional	100	Age: 56.42 ± 6.16 (40–60 years) Sex: M = 72; F = 28 Chronicity: chronic Stroke duration: ≥6 months Stroke type: all ischemic	Fatigue severity scale (FSS) Modified fatigue impact scale (MFIS)	Bathel Index (BI)	Functional recovery	PSF has a significant moderate negative correlation with functional recovery. PSF is prevalent moderately, with 62 and 66% using FSS and MFIS, respectively. PSF severity and impact increase significantly in females.
Bhimani et al., 2022 United States	To determine whether spasticity, pain, and fatigue symptoms were related to functional outcomes in people with stroke.	Longitudinal observational	22	Age: 62 ± 12.7 years Sex: M = 12; F = 10 Chronicity: acute	Numeric rating scale (NRS)	ADL scale, mobility tool, Upper extremity (UE) gross and fine motor tools, lower extremity (LE) motor function (MF) and physical function (PF) tools, Functional independence measure (FIM)	Activity, mobility, upper extremity gross and fine functions (motor), lower extremity functions (motor), physical functioning, independent functions, spasticity, pain.	Fatigue (FG) has a significantly moderate positive correlation with activity (ADLS). FG also has a significantly strong negative correlation with mobility, LE function, and physical function. FG also has a significant moderate negative correlation with UE gross and fine motor functions at 1 month. FG has a significant positive correlation with some dressing and tiredness domains of ADLs at 1 month. The pain was related to fine motor activity on admission. Spasticity was significantly correlated with ADLs, pain, and fatigue at 1 month.
Chen et al., 2015 China	To investigate the risk factors of poststroke fatigue and its effect on activities of daily living and health-related quality of life in Chinese patients with ischemic stroke.	Cross-sectional	218	Age: 61.2 ± 11.4 years Sex: M = 73.4% Chronicity: acute Stroke subtype: Large artery(86), small artery(108), cardioembolism(7), unknown(17) Location of infarct: Cortical region(56), subcortical white matter(92), basal ganglia and thalami(59), infratentorial(51)	FSS	The Hamilton Depression Rating Scale, the Lawton Activities of Daily Living Scale, and the Stroke-Specific Quality of Life Scale.	NIHSS, ADLs, stroke-specific quality of life (SSQOL), depression, hypertension, diabetes mellitus	FSS has a significantly weak correlation with NIHSS scores at admission. FSS has significant moderate positive and strong negative correlations with both ADL and SSQOL (*p* < 0·001), respectively. HDRS, HAS, pre-stroke FG, and current use of antidepressants have a significant correlation with FSS (*p* < 0·05)
Drummond et al., 2017 United Kingdom	To identify factors associated with post-stroke fatigue in a sample of stroke survivors without depression	Cross-sectional cohort	268	Age: 67.7 ± 13.5 years Sex: M = 168, F = 100 Chronicity: acute and subacute Stroke duration: 4–6 weeks Stroke type: Infarction(243), Hemorhhagic:23 Missing: 2 Hemisphere: Right:141, Left:112, bilatral:10, missing:2	FSS of fatigue assessment inventory	The Rivermead Mobility Index, the Nottingham Extended Activities of Daily Living scale, the Beck Anxiety Index (BAI), the Sleep Hygiene Index (SHI), a 6-m walk test(6MWT), and measures of cognitive ability.	Mobility and ADL, mood and emotional factors, cognitive abilities	Fatigue has a significantly weak negative correlation with mobility and a very weak negative correlation with Nottingham scale ADL and Bathel Index ADL. Fatigue also has a positive correlation with emotional factors and sleep hygiene. Fatigue also has a negative correlation with most domains of cognitive abilities.
Gbiri et al., 2020 Nigeria	To determine the prevalence of post-stroke fatigue and explore the inter-relationship between it and the cardiopulmonary functions and quality of life of stroke survivors	Cross-sectional	54	Age: ≥26 years Sex: M = 29, F = 25	FSS	The Stroke-Specific Quality of Life Questionnaire (SSQOL). Spirometer, blood pressure measuring apparatus	Quality of life, cardiopulmonary parameters	FSS has a significantly strong negative correlation with the overall quality of life and a moderate negative correlation with UE function, mobility, energy, family roles, language, personality, and self-care components of SSQOL. However, the correlation between FSS and each of the mood and work productivity components of SSQOL was significantly weak and negative. However, there was no significant correlation between FSS and selected cardiopulmonary parameters.
Goh and Stewart, 2019 United States	To examine the relationship between PSF and motor and cognitive performance using a comprehensive set of behavioral measures and excluding individuals with depression	Cross-sectional	53	Age: 63 years (median) Sex: M = 37, F = 16 Chronicity: chronic Post-stroke duration-median (IQR): 19.5 (6.8–55.0) months Stroke location: Cortical(3), Subcortical(26) Both(6) Lesion hemisphere: Right:29, Left:24	FSS	Montreal Cognitive Assessment (MOCA), simple and choice reaction time (SRT and CRT) tasks, FMA, five-times-sit-to-stand test (5 × STS), the Berg Balance Scale (BBS), Functional Ambulation Category (FAC), and gait speed, grip strength, and the Box and Block test	Cognitive performance, LE, and UE motor performance.	FSS has a significantly weak negative correlation with BBS and FAC. FSS also has significant negative and positive correlations with MOCA assessment scores and SRT/CRT, respectively.
Harmsen et al., 2017 The Netherlands	To evaluate the knee muscle strength and fatigue in patients with aneurysmal subarachnoid hemorrhage (a-SAH)	Prospective observational	33	Age: 52.5 ± 9.5 years Sex: M = 10, F = 23 Chronicity: chronic Duration: 6 months after onset Lesion location: Anterior circulation:18 Posterior circulation:15	FSS	Biodex Dynamometer (Biodex; Shirley, New York, USA)	Knee muscle strength	FSS has a significant moderate negative correlation with extension PT 60°/s and flexion PT 60°/s, while FSS has a significant weak negative correlation with extension PT 180°/s and flexion PT 180°/s. FSS also has a significant correlation with peak torque normalized for body mass and fat-free mass. Knee muscle strength was considerably lower in patients with a-SAH than in matched controls. Measures of knee muscle strength were 21–40% lower in patients than in control. Deficits in muscle strength may play a role in fatigue.
Ho et al., 2021a Hong Kong	To determine the prevalence of fatigue and poor sleep quality and to quantify the contribution of sleep quality to fatigue following a stroke in chronic (≥1 year) stroke survivors	Cross-sectional	112	Age: 64.18 ± 5.77 years Sex: M = 74, F = 38 Chronicity: chronic Time since the last stroke: 6.08 ± 4.80 years	Fatigue Assessment Scale (FAS)	The Pittsburgh Sleep Quality Index (PSQI), the VAS-Pain, FMA-UE and FMA-LE, the 5-Time Sit-To-Stand Test (FTSTS), the Epworth Sleepiness Scale (ESS), the Frenchay Activities Index (FAI), the Life-Space Assessment (LSA), the Community Integration Measure (CIM), and the Multidimensional Scale of Perceived Social Support (MSPSS)	Sleep quality, pain, motor control, LE functional muscle strength, daytime sleepiness, frequency of participation in activities, environmental and personal factors affecting mobility, community integration, social support	FAS has a significant, very weak positive correlation with FMA-UE and FMA-LE. FAS has a significantly weak negative correlation with participation in activities and community integration. FAS has no significant, very weak negative correlation with LSA. FAS also correlates positively with PSQI and ESS and negatively with MSPSS. The prevalence of fatigue was 52.7, and 64.3% for poor sleep quality. Sleep quality was an independent, significant predictor of fatigue.
Ho et al., 2021b Hong Kong	To translate and adapt the Fatigue Assessment Scale into Chinese, examine its psychometric properties, determine the levels of physical and mental fatigue, and compare the fatigue scores in stroke survivors with and without depressive symptoms	Cross-sectional	112	Age: 64.15 ± 5.79 years Sex: M = 74, F = 38 Chronicity: chronic Time since stroke: 6.13 ± 4.79 years	FAS	FSS, mental fatigue scale (MFS). FMA-UE and FMA-LE, FTSTS, GDS, ESS,	Fatigue, mental fatigue, physical performance, functional muscle strength, depression, and sleepiness	FAS has a significantly strong positive correlation with MFS and a significant moderate positive correlation with FSS. However, FAS has a significantly weak correlation with FAM-UE, FMA-LE, and ESS but not with FTSTS. CFAS is a valid and reliable tool for fatigue assessment. Physical fatigue was more than mental fatigue in the participants, and those with depressive symptoms had higher fatigue scores.
Hubacher et al., 2012 Switzerland	To characterize PSF beyond the subacute phase.	Cross-sectional	31	Age: 59.29 ± 10.30 years Sex: M = 25, F = 6 Chronicity: chronic Stroke type: Ischemic:128 Hemorhhagic:3 Stroke localization: Cortical(C):6, subcortical(SC):19 Cortical and subcortical:6 Hemisphere Right hemisphere: 15 Left hemisphere: 11 Both hemispheres:3 Unknown:2	FSS, MFIS, FSMC	Brief Repeatable Battery of Neurophysiological Tests (BRB-N), the Nine-Hole Peg Test (9-HPT), 25-foot walk measure, Bech Depression Inventory (BDI)	Memory, processing speed, executive functions, UE functions, LE functions, depression	FSS has no significant positive or negative correlation with UE functions and LE functions. FSS also has no significant correlation with memory, mental speed, or executive functions. However, some components of MFIS AND FSMC have shown significant correlation with some aspects of memory, UE, and LE functions.
Lewis et al., 2011 United States	To determine the relationship between a measure of fatigue and two indices of physical fitness, lower limb extensor power (LLEP) and walking economy	Cross-sectional	66	Age: 71.10 ± 9.9 years Sex: M = 36, F = 30 Chronicity: chronic Median time interval from stroke onset to assessment: 160 days (IQR, 84-280d)	Vitality component of SF-36v2 (VITs), mental health score of SF-36v2 (MH), and role emotional score of SF-36v2(RH)	lower leg extensor power rig, oxygen consumption (mL·kg^−1^·m^−1^) during walking at a comfortable speed	Lower limb extensor power (LLEP), walking economy	VIT has a significantly weak positive correlation with LLEP. The walking economy was not significantly related to VIT. MH and RE were strongly intercorrelated and associated individually. A higher LLEP score and the unaffected limb were associated with a higher VIT score.
Mahendran et al., 2020 Australia	To determine which impairments, activity limitations, and personal factors at hospital discharge poststroke predict volume, frequency, and intensity of walking activity 1, 3, and 6 months late	Prospective longitudinal observational	36	Age: 71 ± 14 years Sex: M = 25(69%), F = 11(31%) Chronicity: subacute Duration: Stroke within the past 4 months Side affected: right hemiplegia: 21	FSS	Physical Activity Scale for the Elderly (PASE), the Ambulatory Self-confidence Questionnaire (ASCQ), Stroke Impact Scale (SIS), European Quality of Life-5 D (EQ-5D), ActivPAL™, 6 MWT	Mood, executive function, walking speed, walking endurance, age, pre-stroke activity, self-efficacy, and perceived stroke recovery and health were collected. Walking activity and volume of walking activity	FSS has a significant weak negative correlation with the volume of steps and a moderate negative correlation with long bouts and high-intensity bouts at 1 month. Across 6 months after hospital discharge, walking endurance contributes to walking activity outcomes. Following 1 month of discharge, factors other than post-stroke changes also contribute to activity outcomes and should be taken into consideration when aiming at post-stroke physical activity.
Mandliya et al., 2016 India	To examine the role of PSF in post-stroke disability and burden of care among stroke survivors after their first-ever stroke	Prospective cross-sectional	163	Age: 71.10 ± 9.9 years Sex: M = 128, F = 35 Chronicity: subacute and chronic Post-stroke duration: median: 6.7 months, range (3–64 months) Stroke type: Ischemic:146 Hemorhhagic:17 Side affected: Right:76 Left:81	SF-36 vitality domain (VITs)	The Modified Rankin Scale, the Hospital Anxiety and Depression Scale, Functional Independence measure, NIHSS	Functional recovery, depression, disability and burden of care, stroke severity	VITs have a significantly weak positive correlation with disability/burden of care. Disability/burden of care also has a significant negative correlation with functional recovery and depression. The severity of PSF has a positive correlation with disability. The PSF has a significant contribution to disability over and above functional recovery and depression. There is an independent negative impact of PSF on the disability of post-stroke patients.
Michael et al., 2006 United States	To quantify fatigue in a sample of individuals with chronic hemiparetic stroke and to explore the relationships of fatigue to cardiovascular fitness, mobility deficit severity, ambulatory activity patterns, social support, and self-efficacy for falls	Cross-sectional	53	Age: 45–84 years(mean = 66) Sex: M = 31, F = 22 Chronicity: Chronic Time after stroke: 6–166 months (mean = 10.3)	FSS	Timed 10-meter walks, the Berg Balance Scale, submaximal and peak VO2, total daily step activity derived from microprocessor-linked Step Activity Monitors, the Medical Outcomes Study Social Support Survey, and the Falls Efficacy Scale	Mobility deficit severity, cardiovascular-metabolic fitness, ambulatory activity, social support, and self-efficacy for fall	FSS has a significant moderate negative correlation with BBS and a moderate positive correlation with FES, but not with ambulatory activity or fitness. VAS has a significant positive correlation with FSS but not with social support. Only FES significantly predicts FSS. BBS is the key predictor of ambulatory activity. Patients with increased FSS scores had low social support.
Michael and Macko, 2007 United States	To describe household and community ambulatory activity profiles and their relationship to fatigue and cardiovascular fitness in a sample of men and women with chronic hemiparetic stroke.	Cross-sectional	79	Age: 45–84 years(mean = 65) Sex: M = 42, F = 37 Chronicity: Chronic Time post stroke: 6–166 months (mean = 10.3)	FSS	Step Activity Monitors (SAMs) (Cymatech; Cyma Corporation, Seattle, WA), treadmill testing at submaximal effort with open-circuit spirometry	Community-based ambulatory activity profiles in terms of step counts and intensity, cardiovascular fitness, gait economy	FSS has no significant positive correlation with step/ambulatory activity. Participants show extremely low step counts, with almost no stepping activity at high intensity. Stepping activity intensity has a strong correlation with cardiovascular fitness.
Miller et al., 2013 USA	To examine the frequency and impact of fatigue and pain in people with chronic stroke	Prospective cross-sectional	77	Age: 48–89 years (mean = 64.1) Sex: M = 58, F = 19 Chronicity: Chronic Time since stroke: > 6 months since stroke Stroke type: Ischemic:34 Hemorrhagic: Lesion side: Right:30 Left:	FSS	Brief Pain Inventory (BPI), BBS, 6 MWT, 10MWT, ICF Measure of Participation and Activities (IMPACT), Chronic Disease Self-Efficacy (CDSE) scale, and the Activities-Specific Balance Confidence (ABC) scale	Pain, balance, gait (waking distance and gait speed), balance, activity and participation, self-efficacy	FSS has a significant negative moderate correlation with CDSE and ABC. The FSS also has a significantly weak negative correlation with BBS and 6 MWT. FSS also has a significantly moderate and positive correlation with IMPACT-Activity and a significant weak positive correlation with IMPACT-Participation. FSS has no significant correlation with 10MWT. Pain is correlated with CDSE, ABC, activity, and participation. Fatigue and pain are common post-stroke, and they correlate negatively with outcomes important to rehabilitation. Fatigue was reported in 66% of the participants, and 34% reported co-existing fatigue and pain.
Muci et al., 2022 Turkey	To investigate the relationships between dual-task performance and factors such as motor function, balance, cognitive state, and fatigue to identify factors that have an impact on dual-task performance after a stroke.	Cross-sectional	37	Age: 54.03 ± 13.50 years Sex: M = 25, F = 12 Chronicity: subacute and chronic Post-stroke duration: 4 (1–45) months Lesion type: Ischemic:28 Hemorhhagic:9 Hemiparetic side: Right:21 Left:16	FSS	10-m walking test in different performances, which were single-task-based, motor dual-task-based, and cognitive dual-task-based. The Rivermead Motor Assessment (RMA), the Static Balance Index (SBI) using Kinesthetic Ability Trainer 3000 (KAT) (Med-Fit Systems Inc., Fallbrook, CA, USA), and Mini-Mental State Examination (MMSE)	Gait speed, single task performance (STP), motor dual-task performance (MDTP), cognitive dual-task performance (CDTP), motor function (i.e., gross, leg-trunk, and arm), mobility, static balance and post-stroke duration (PSD)	FSS has a significantly strong positive correlation with SBI and a significantly weak negative correlation with STP, MDTP, and CDTP. FSS has no significant correlation with age, PSD, or MMSE.
Obembe et al., 2014 Nigeria	To explore the relationship between functional limitations due to post-stroke fatigue with gait and balance performance in stroke survivors undergoing physiotherapy.	Cross-sectional	70	Age: 54.3 ± 8.5 (42-76)years Sex: M = 41, F = 29 Chronicity: Chronic Stroke duration: 12.4 ± 7.6 (6–36) months Stroke type: Ischemic:38 Hemorhhagic:32 Side affected: Right:45 Left:25	Modified fatigue impact scale (MFIS)	Observational gait analysis, BBS, FES	Gait speed, cadence, balance performance, fall efficacy	MFIS has no statistically significant positive correlation with gait speed, cadence, and fall efficacy, with no significant negative correlation with balance performance. A total of 37.1% of the participants often experienced functional limitations due to fatigue.
Obembe et al., 2015 Nigeria	To assess the functional limitations due to fatigue in community-dwelling stroke survivors undergoing physiotherapy	Cross-sectional	63	Age: 45–79 years (53.68 ± 10.95 years) Sex: M = 35, F = 28 Chronicity: chronic stroke duration: 15.88 ± 4.81 months Stroke type: Ischemic:36 Hemorhhagic:27 Side affected: Right:38 Left:25	The Modified Fatigue Impact Scale (MFIS)	The Modified Rankin Scale (MRS)	Disability	MFIS has a significantly strong positive correlation with the level of disability (MRS). MFIS has a weak negative correlation with stroke type. The level of disability and sex significantly determine the impact of fatigue. A total of 58.7% of the participants had moderate functional limitations due to fatigue. A higher MIF score was found in participants with moderate disability. Functional limitation due to fatigue significantly differs between those with slight and moderate disability.
Park et al., 2009 Korea	To evaluate the influence of fatigue on functional outcomes after stroke	Cross-sectional	40	Age: 59.9 ± 11.8 years Sex: M = 26 (65%), F = 35% Chronicity: Chronic Post-stroke duration: 32.7 ± 27.4 months Stroke type: Ischemic:62.5% Hemorrhagic: Side affected: Right:30% Left:60%	FSS	The Modified Barthel Index (MBI), the Motricity Index (MI), and the Korean-Mini Mental State Examination (K-MMSE). The Beck Depression Inventory (BDI) was interviewed.	The activity of daily living (ADL) function, motor function, cognitive function, depression, sleeping problems	FSS has a significantly moderate positive correlation with BDI. It also has a significant correlation with sleep problems but no significant correlation with MBI, MI, and K-MMSE. Moderate to high fatigue is not unusual poststroke. A total of 30% of participants had post-stroke fatigue, 55% had depression, and 30% had sleep problems.
Pedersen et al., 2022 Sweden	To explore predictive and cross-sectionally correlated features in the long term after ischemic stroke	Prospective longitudinal follow-up	430	Age: median(1QR) 57 (49–63) years Sex: M = 282, F = 148 Chronicity: Chronic	FIS and daily-FIS	The Scandinavian Stroke Scale (SSS), NIHSS, Saltin-Grimby Physical Activity Level Scale (SGPALS), Barrow Neurological Institute Screen for Higher Cerebral Functions (BNIS), Mrs. Karolinska Sleep Questionnaire (KSQ), HADS, short form-36 (SF-36)	Index stroke severity, physical exercise, cognitive function, functional outcome, neurological impairment, insomnia, HADS, SF-36	D-FIS has a significant positive moderate correlation with mRS, HADS-D, and HADS-A. The D-FIS has a significant negative, weak correlation with NIHSS and a moderate negative correlation with SF-36 bodily pain. D-FIS has a significantly weak positive correlation with sedentary lifestyle and insomnia and a very weak positive correlation with recurrent stroke. D-FIS has no significant, very weak correlation with living with a partner.
Pedersen et al., 2023 Norway	To investigate the independent contribution of 12 function-related domains to severe long-term fatigue	Observational follow-up	144	Age: 67.3 ± 10.9 years Sex: M = 92, F = 52 Chronicity: Chronic Stroke type: Ischemic: 130 Hemorrhagic: 14	FSS	Multidimensional Stroke-Specific Quality of Life (SS-QOL) scale	Function-related consequences	FSS has a significantly strong negative correlation with overall SSQOL score, mood, energy, and cognitive-social-mental component. Moreover, FSS has a significantly moderate negative correlation with mobility, work/productivity, UE function, thinking, personality, family roles, social roles, and physical health components. FSS has a significantly weak negative correlation with self-care, vision, and language.
Rahamatali et al., 2021 Belgium	(i) To evaluate the prevalence of perceived fatigue and fatigability amongst patients with chronic stroke and to explore how these two parameters relate. (ii) To study the relationship between modifiable factors (sleep disorders, anxiety, depression, and activities of daily living) and fatigue in this population	Cross-sectional	62	Age: 59 ± 11 years Sex: M = 37, F = 25 Chronicity: Chronic Time since stroke: 4 ± 3.5 years Stroke type: Ischemic: Hemorrhagic: Side affected: Right, Left	FSS	6-min Walk Test (6MWT), isometric muscular fatigue test with the Neuromuscular Fatigability Index (NMFI), the Hospital Anxiety and Depression Scale (HADS), the Pittsburgh Sleep Quality Index (PSQI), and ACTIVLIM-stroke	Motor fatigability, anxiety and depression, sleep quality, sleep quality, activity limitations	FSS has a significantly weak negative correlation with ACTIVLIM but did not have a significant correlation with motor fatigability (6MWT and NMFI). FSS has a moderately positive correlation with HADS-A. HADS-D, PSQI, and ACTIVLIM significantly determine FSS. A total of 71% patients had PSF.
Schow et al., 2017 Denmark	To investigate fatigue after stroke and its relation to balance, gait, and binocular visual dysfunction (BVD)	Cross-sectional	29	Age: 52,86 ± 7,5 years Sex: M = 16, F = 13 Chronicity: Subacute and chronic Time since injury: 5,24 ± 3,56 months Injury Localization: Brainstem:4, cerebellum:7, right:3, left: 8, bilateral: 7	MFIS	EuroQol five-dimension three level measure (EQ-5D-3L), the Vertical Heterophoria Symptom Questionnaire (VHS-Q), Randot Sterotest (Stereo Optical Co., Chicago, IL), King-Devick test (K-D), Wolff Wand, Keystone Telebinocular, The BESTest and 10MWT	Health-related quality of life, BVD burden, stereo acuity at Near (SAN), reading-related saccadic eye movements, Near Point of Convergence (NPC), Binocular fusion, balance and gait, gait performance	MFIS cognitive (MFISC) has a significant correlation with walking and reactive postural control, and MFIS arousal (MFISA) has a significant correlation with reactive postural control, postural adjustment, and gain stability. MFIS physical (MFISP) and HRQOL have no significant correlation with balance or walking speed. Arousal was majorly related to dizziness, pain, K-D, MIFSP is strongly related to vision, in VHSQ pain/sensation, VHSQ vision, BFN, convergence (NPC), and TBI-MFIS-physical is associated with dizziness and pain. HRQOL is associated with BFN and NPC. Time in the treatment program is the strongest predictor of post-stroke fatigue
Stookey et al., 2021 United States	To establish test–retest reliability of fatigability in stroke during 6-min walk (6 MW) testing. Relationships between post-stroke fatigability and other constructs were assessed.	Cross-sectional	23	Age: 61 ± 8.5 (41–80) years Sex: M = 12; F = 11 Chronicity: chronic Stroke duration: >6 months	VAS	Open circuit spirometry, screening treadmill tolerance test, 30-ft walk tests, 6 MWT, the Dynamic Gait Index (DGI), step activity monitors, Falls efficacy scale	6 MW oxygen consumption, peak aerobic capacity (VO_2_ peak), gait speed, including self-selected walking speed (SSWS) and fastest comfortable walking speed (FCWS), balance, fall risk, ambulatory activity, fall self-efficacy/self-confidence.	VAS fatigue has no significant positive or negative correlations with FES and performance fatigability (PF).VAS has no significant negative correlation with BMI. PF has a significantly strong negative correlation with SSWS, FCWS, DGI, and gait speed 6 MW. PF has a significant, very strong positive correlation with VO2 6 MW. PF also has a significant moderate negative correlation with VO_2_ peak, and PF has no significant correlation with step count.
Tseng and Kluding, 2009 United States	To explore the relationship between fatigue, aerobic fitness, and motor control in people with chronic stroke	Cross-sectional	9	Age: 56.8 ± 11.8 (47–73) years Sex: M = 2; F = 7 Chronicity: chronic Time post-stroke duration: 47.6 ± 51.2 (11–140) months	The Fatigue Index Scale (FI) to report fatigue at the moment/state of fatigue	6MWD, cycle-eargometer, Fugl-Meyer (FM) test,	Aerobic fitness in the form of VO_2peak_, motor control,	FI has a significantly negative and strong correlation with VO_2peak_ and FM, but not with 6 MWD. VO_2peak_ has a significantly strong positive correlation with FM and 6 MWD. Motor control is an independent predictor of fatigue.
Tseng et al., 2010 United States	To identify the contributing factors of exertion fatigue and chronic fatigue in people post-stroke	Cross-sectional	21	Age: 59.5 ± 10.3 years Sex: M = 12; F = 9 Chronicity: chronic Time after stroke: 4.1 ± 3.5 years Stroke type: Ischemic:18, Hemorhhagic:3 Lesion side: Right:15, Left:4, Brain stem:2	Visual Analog Fatigue Scale (VAFS), FSS	Peak oxygen uptake (VO_2peak_), Fugl-Meyer motor score (FMA), and the Geriatric Depression Scale (GDS)	Aerobic fitness, motor control, and depressive symptoms	Exertion fatigue (EF) has significant moderate correlations with VO_2peak_. Chronic fatigue has a strong positive correlation with GDS and VAFS_at rest_. VO_2peak_ has a significantly strong positive correlation with recovery rate (RR). Exertion and chronic fatigue have negative and positive non-significant correlations with motor function, respectively.
van der Werf et al., 2001 Netherlands	To test whether the experience of severe fatigue persists long after a stroke has occurred and to assess the relation between experienced fatigue and levels of physical impairment and depression	Cross-sectional	90	Age: 62.1 (32–73) years Sex: M = 65; F = 25 Chronicity: chronic Median time since stroke: 2 years	The Checklist Individual Strength (CIS) Fatigue Scale	BDI primary care (BDI-PC), the Sickness Impact Scale (SIP),	Depression, functional disability, SIP ambulation, SIP alertness behavior, Neuropsychological and physical impairment	CIS has a significantly strong positive correlation with perceived disability. Time since stroke has no significant correlation with CIS fatigue, BDI-PC, SIP ambulation, SIP alertness behavior, and SIP7-total. SIP ambulation explains 34% of CIS-F.

### Assessment of the methodological quality

The National Institute of Health (NIH) 14-item Quality Assessment Tool for Observational Cohort and Cross-Sectional Studies (22) was used to evaluate the internal validity and risks of bias in measurement, information, selection, or confounding in the included studies. This tool also covers aspects such as study design, population, sample size, attrition, blinding, data collection, outcomes, and outcome measures. Each item is rated as ‘Yes’ or ‘No,’ with room for selecting other options such as ‘cannot be determined’ (CD), ‘not reported’ (NR), or ‘not applicable’ (NA). An overall quality appraisal rating of good, fair, or poor is made for each evaluated article (Table 2). The quality assessments were independently performed by two researchers (JSU and TWLW). Any disagreements between the two researchers were resolved by consulting a third researcher (SSMN).

**Table 2:** Methodological quality of the included studies according to the NIH quality assessment tool for observational cohort and cross-sectional studies.

Study	Appraisal criteria of the NIH 14-item quality assessment tool														Score	Quality	Findings
Author year	1	2	3	4	5	6	7	8	9	10	11	12	13	14	Total Score (14)	Overall quality rating	Quality appraisal findings
Ali 2022	✓	✓	✓	✓	X	X	X	NA	✓	X	✓	NA	NA	X	6	Fair quality	Cross-sectional study, no sample size justification, small sample size, exposure and outcomes measures at the same time, no time to see effect, exposure assessed once, no blinding, and no confounders were adjusted statistically, the validity and reliability of the measures of exposure and some outcomes were stated
Bhimani 2022	✓	✓	NR	✓	NA	X	X	X	✓	✓	✓	X	NR	X	6	Fair quality	Longitudinal correlational, no sample size justification, small sample size, exposure and outcomes measured at the same time, no cause and effect, no blinding, and no confounders were adjusted statistically, exposure and outcomes were measured more than once, and the validity and reliability of the measures of exposure and outcomes were stated
Chen 2015	✓	✓	✓	✓	X	X	X	NA	✓	X	✓	✓	NA	X	7	Fair quality	Cross-sectional study, no sample size justification, small sample size, exposure and outcomes measures at the same time, no cause and effect, exposure assessed once, and no confounders were adjusted statistically, there was blinding, validity, and reliability of the measures of exposure, and outcomes were stated
Drummond 2017	✓	✓	✓	✓	✓	X	X	NA	✓	X	✓	X	NA	X	7	Fair quality	Cross-sectional cohort, exposure and outcomes measures at the same time, no cause and effect, exposure assessed once, no blinding, and no confounders were adjusted statistically, the sample size was justified, but the validity and reliability of the measures of exposure and outcomes were not stated
Gbiri 2020	✓	✓	✓	✓	X	X	X	NA	✓	X	✓	X	NA	X	6	Fair quality	Cross-sectional study, no sample size justification, small sample size, exposure and outcomes measures at the same time, no cause and effect, exposure assessed once, no blinding, and no confounders were adjusted statistically, the psychometric properties of some of the measures of outcomes and exposure were not stated, no recruitment time specification and no specific name of centers for recruitment.
Goh 2019	✓	✓	✓	✓	X	X	X	NA	✓	X	✓	X	NA	X	6	Fair quality	The cross-sectional observational sample size was not justified. Small sample size, exposure and outcomes measures at the same time, no cause and effect, exposure was assessed once, no blinding, no confounders were adjusted for, the validity of the measures of exposure was stated but not for measures of outcomes, and the specific name of the center for recruitment was not stated
Harmsen 2017	✓	✓	✓	✓	X	X	X	NA	✓	X	✓	X	NA	✓	7	Fair quality	In the cross-sectional study, the sample size was not justified, small sample size, exposure and outcomes were measured at the same time, no cause and effect, exposure was assessed once, no blinding, confounders were adjusted statistically, and the validity of a measure was stated, the inclusion criteria were not very explicit
Ho 2021a	✓	✓	✓	✓	✓	X	X	NA	✓	X	✓	X	NA	✓	8	Fair quality	Cross-sectional study, exposure and outcomes were measured at the same time, with no cause and effect. Exposure was assessed once, no blinding, and confounders were adjusted statistically, the psychometric properties of the measures of exposure and outcomes were stated, and the sample size was justified.
Ho 2021b	✓	✓	✓	✓	✓	X	X	NA	✓	✓	✓	X	NA	X	8	Fair quality	Cross-sectional study, exposure and outcomes measures at the same time, no cause and effect, exposure assessed once, no blinding, and no confounders were adjusted statistically, exposure and outcomes were measured more than once, and the psychometric properties of the measures of exposure and outcomes were stated, and the sample size was justified
Hubacher 2012	✓	✓	✓	✓	X	X	X	NA	✓	X	✓	X	NA	X	6	Fair quality	Cross-sectional study, the sample size was not justified, small sample size, exposure and outcomes measures were taken at the same time, no cause and effect, exposure assessed once, no blinding, and no confounders were adjusted statistically, the psychometric properties of the measures of exposures and outcomes were not stated
Lewis 2011	✓	✓	✓	✓	X	X	X	NA	✓	X	✓	X	NA	X	6	Fair quality	Cross-sectional study, no sample size justification, small sample size, exposure and outcomes measures at the same time, no cause and effect, exposure assessed once, no blinding, confounders were adjusted statistically, the psychometric properties of the measures of exposure and outcomes were stated, the exclusion criteria not stated, the specific name of the center for recruitment not stated
Mahendran 2020	✓	✓	✓	✓	✓	X	✓	NA	✓	✓	✓	X	X	X	9	Fair quality	Prospective longitudinal, no sample size justification, small sample size, exposure and outcomes measures at the same time, no blinding, and no confounders were adjusted statistically, exposure and outcomes were measured more than once, the validity and reliability of the measures of exposure and outcomes were not stated
Mandliya 2016	✓	✓	✓	✓	X	X	X	NA	✓	X	✓	X	NA	✓	7	Fair quality	Prospective cross-sectional, no sample size justification, small sample size, exposure and outcomes measures at the same time, no time to see the effect, exposure assessed once, no blinding, confounders were controlled for, the psychometric properties of some of the measures of exposure and outcomes were stated,
Michael 2006	✓	✓	✓	✓	X	X	X	NA	✓	X	✓	X	NA	X	6	Fair quality	Cross-sectional study, no sample size justification, small sample size, exposure and outcomes measures at the same time, no cause and effect, exposure assessed once, no blinding, and some confounders were measured, and the psychometric properties of most of the measures of outcomes were stated but not for exposure, no recruitment time specification
Michael 2007	✓	✓	✓	✓	X	X	X	NA	✓	X	✓	X	NA	X	6	Fair quality	Cross-sectional study, no sample size justification, small sample size, exposure and outcomes measures at the same time, no cause and effect, exposure assessed once, no blinding, and no confounders were adjusted statistically, the psychometric properties of some of the measures of outcomes were stated, no recruitment time specification
Miller 2013	✓	✓	✓	✓	X	X	X	NA	✓	X	✓	X	NA	X	6	Fair quality	Cross-sectional study, no sample size justification, small sample size, exposure and outcomes measures at the same time, no cause and effect, exposure assessed once, no blinding, and no confounders were adjusted statistically, and the psychometric properties of the measures of outcomes and exposure were stated, with no specification of location and time of recruitment,
Muci 2022	✓	✓	✓	✓	✓	X	X	NA	✓	X	✓	X	NA	X	6	Fair quality	Cross-sectional study, no sample size justification, small sample size was justified, exposure and outcomes measures at the same time, no cause and effect, exposure assessed once, no blinding, no confounders were adjusted statistically, and the psychometric properties of the measures of outcomes and exposure were stated
Obembe 2014	✓	✓	✓	✓	X	X	X	X	✓	X	✓	X	NA	X	6	Fair quality	Cross-sectional study, no sample size justification, small sample size, exposure and outcomes measures at the same time, no cause and effect, exposure assessed once, no blinding, and no confounders were adjusted statistically, the psychometric properties of the measures of outcomes and exposure were not stated, and no specification of the recruitment period
Obembe 2015	✓	✓	✓	✓	X	X	X	NA	✓	X	✓	X	NA	X	6	Fair quality	Cross-sectional study, no sample size justification, small sample size, exposure and outcomes measures at the same time, no cause and effect, exposure assessed once, no blinding, and no confounders were adjusted statistically, and the psychometric properties of the measures of outcomes and exposure were not stated
Park 2009	✓	✓	✓	✓	X	X	X	NA	✓	X	✓	X	NA	X	6	Fair quality	Cross-sectional study, no sample size justification, small sample size, exposure and outcomes measures at the same time, no cause and effect, exposure assessed once, no blinding, no confounders were adjusted statistically, no specifications of the name of the recruitment center, the inclusion criteria were not explicit, and the psychometric properties of some of the measures of outcomes were stated
Pedersen 2022	✓	✓	✓	✓	X	X	✓	X	✓	✓	✓	X	✓	X	8	Fair quality	Prospective longitudinal follow-up, no sample size justification, exposure and outcomes measures at the same time, no blinding, and no confounders were adjusted statistically, exposure and outcomes were measured more than once, and the psychometrics properties of the measures of outcomes were stated but not for exposure
Pedersen 2023	✓	✓	✓	✓	X	X	X	NA	✓	X	✓	X	✓	✓	8	Fair quality	Observational follow-up, no sample size justification, small sample size, exposure and outcomes measures at the same time, exposure assessed once, no blinding, confounders were measured, the inclusion criteria were not very explicit
Rahamatali 2021	✓	✓	✓	✓	X	X	X	NA	✓	X	✓	X	NA	X	6	Fair quality	Cross-sectional study, no sample size justification, small sample size, exposure and outcomes measures at the same time, no cause and effect, exposure assessed once, no blinding, no confounders were adjusted statistically, no specifications of the name of the recruitment center, and the psychometric properties of very few measures of outcomes were stated
Schow 2017	✓	✓	✓	✓	X	X	X	✓	✓	X	✓	X	NA	X	7	Fair quality	Cross-sectional study, no sample size justification, small sample size, exposure and outcomes measures at the same time, no cause and effect, exposure assessed once, no blinding, no confounders were adjusted statistically, no specifications of the name of the recruitment center, no specification of the recruitment period, and the psychometric properties of outcomes measures were not stated
Stookey 2021	✓	✓	✓	✓	X	X	X	NA	✓	X	✓	X	NA	X	6	Fair quality	Cross-sectional study, no sample size justification, small sample size, exposure and outcomes measures at the same time, no cause and effect, exposure assessed once, no blinding, no confounders were adjusted statistically, no specifications of the name of recruitment location and center, no specification of the recruitment period, and the psychometric properties of exposure and outcomes measures were not stated
Tseng 2009	✓	✓	✓	✓	X	X	X	NA	✓	X	✓	X	NA	X	6	Fair quality	Cross-sectional study, no sample size justification, small sample size, exposure and outcomes measures at the same time, no cause and effect, exposure assessed once, no blinding, no confounders were adjusted statistically, no specifications of the name of recruitment location and center, no specification of the recruitment period, and the psychometric properties of outcomes measures were stated
Tseng 2010	✓	✓	✓	✓	X	X	X	NA	✓	X	✓	X	NA	X	6	Fair quality	Cross-sectional study, no sample size justification, small sample size, exposure and outcomes measures at the same time, no time to see effect, exposure assessed once, no blinding, and no confounders were adjusted statistically, validity and reliability of the measures of exposure and outcomes were stated, no time of recruitment was specified
van der Werf 2001	✓	✓	✓	✓	✓	X	X	NA	✓	X	✓	X	NA	X	7	Fair quality	Cross-sectional study, no sample size justification, small sample size, exposure and outcomes measures at the same time, no time to see effect, exposure assessed once, no blinding, and no confounders were adjusted statistically, the validity and reliability of the measures of exposure and outcomes were not stated, no time of recruitment was specified, and the inclusion and the exclusion criteria were not explicitly described

NIH: National Institute of Health, ✓: Yes, X: No, NA: Not applicable, NR: Not reported, Items/Questions: 1. Was the research question or objective in this paper clearly stated? 2. Was the study population clearly specified and defined? 3. Was the participation rate of eligible persons at least 50%? 4. Were all the subjects selected or recruited from the same or similar populations (including the same time period)? Were the inclusion and the exclusion criteria for being in the study prespecified and applied uniformly to all participants? 5. Was a sample size justification, power description, or variance and effect estimates provided? 6. For the analyses in this paper, were the exposure(s) of interest measured prior to the outcome(s) being measured? 7. Was the timeframe sufficient so that one could reasonably expect to see an association between exposure and outcome if it existed? 8. For exposures that can vary in amount or level, did the study examine different levels of the exposure as related to the outcome (e.g., categories of exposure or exposure measured as a continuous variable)? 9. Were the exposure measures (independent variables) clearly defined, valid, reliable, and implemented consistently across all study participants? 10. Was the exposure(s) assessed more than once over time? 11. Were the outcome measures (dependent variables) clearly defined, valid, reliable, and implemented consistently across all study participants? 12. Were the outcome assessors blinded to the exposure status of participants? 13. Was loss to follow-up after baseline 20% or less? 14. Were key potential confounding variables measured and adjusted statistically for their impact on the relationship between exposure(s) and outcome(s)?

### Assessment of risk of bias (ROB)

The ROB of the included studies was evaluated using the Quality in Prognosis Studies (QUIPS) checklist (23). The QUPS checklist assesses ROB across six domains, namely study participation, study attrition, prognostic factor measurement, outcome measurement, study confounding, and statistical analysis and reporting (23). Each of the domains for each study, as well as the overall study, is judged as low, moderate, or high in ROB (24). The results of these evaluations are shown in Table 3. The QUIPS checklist was adapted and used to assess ROB in the context of this review, and the details of the scoring and evaluation method used in the adapted QUIPS checklist are contained in Supplementary material S2. During the evaluation, the domain of study attrition was not scored for most of the studies because it was not applicable to them, being cross-sectional in nature. Two researchers (JSU and TWLW) independently performed the assessments. Any disagreements regarding ROB ratings were resolved by consulting a third researcher (SSMN).

**Table 3:** Risk of bias (ROB) in the included studies according to QUIPS ROB tool.

Serial no.	Author year	Study Participation	Study Attrition	Prognostic Factor (fatigue) Measurement	Outcome Measurement	Study Confounding	Statistical Analysis and Reporting	Overall Risk of Bias
1.	Ali 2022	Low risk	Not applicable	Low risk	Low risk	Moderate risk	Low risk	Low risk
2.	Bhimani 2022	Low risk	Low risk	Low risk	Low risk	Moderate risk	Low risk	Low risk
3.	Chen 2015	Low risk	Not applicable	Low risk	Low risk	Moderate risk	Low risk	Low risk
4.	Drummond 2017	Low risk	Not applicable	Low risk	Low risk	Moderate risk	Low risk	Low risk
5.	Gbiri 2020	Moderate risk	Not applicable	Low risk	Low risk	Moderate risk	Low risk	Moderate risk
6.	Goh 2019	Moderate risk	Not applicable	Low risk	Moderate risk	High risk	Low risk	High risk
7.	Harmsen 2017	Low risk	Not applicable	Low risk	Low risk	Low risk	Low risk	Low risk
8.	Ho 2021a	Low risk	Not applicable	Low risk	Low risk	Low risk	Low risk	Low risk
9.	Ho 2021b	Low risk	Not applicable	Low risk	Low risk	Moderate risk	Low risk	Low risk
10.	Hubacher 2012	Low risk	Not applicable	Low risk	Low risk	Moderate risk	Low risk	Low risk
11.	Lewis 2011	Moderate risk	Not applicable	Low risk	Low risk	Low risk	Low risk	Low risk
12.	Mahendran 2020	Low risk	Low risk	Low risk	Low risk	Moderate risk	Low risk	Low risk
13.	Mandliya 2016	Low risk	Not applicable	Low risk	Low risk	Low risk	Low risk	Low risk
14.	Michael 2006	Moderate risk	Not applicable	Low risk	Low risk	Low risk	Low risk	Low risk
15.	Michael 2007	Moderate risk	Not applicable	Low risk	Low risk	Moderate risk	Low risk	Moderate risk
16.	Miller 2013	Moderate risk	Not applicable	Low risk	Low risk	Moderate risk	Low risk	Moderate risk
17.	Muci 2022	Low risk	Not applicable	Low risk	Low risk	Moderate risk	Low risk	Low risk
18.	Obembe 2014	Moderate risk	Not applicable	Low risk	Low risk	Moderate risk	Low risk	Moderate risk
19.	Obembe 2015	Low risk	Not applicable	Low risk	Low risk	Moderate risk	Low risk	Low risk
20.	Park 2009	Moderate risk	Not applicable	Low risk	Low risk	Moderate risk	Low risk	Moderate risk
21.	Pedersen 2022	Low risk	Low risk	Low risk	Low risk	Moderate risk	Low risk	Low risk
22.	Pedersen 2023	Low risk	Low risk	Low risk	Low risk	Low risk	Low risk	Low risk
23.	Rahamatali 2021	Moderate risk	Not applicable	Low risk	Low risk	Moderate risk	Low risk	Moderate risk
24.	Schow 2017	Moderate risk	Not applicable	Low risk	Low risk	Moderate risk	Low risk	Moderate risk
25.	Stookey 2021	High risk	Not applicable	Low risk	Low risk	Moderate risk	Low risk	High risk
26.	Tseng 2009	Moderate risk	Not applicable	Low risk	Low risk	Moderate risk	Low risk	Moderate risk
27.	Tseng 2010	Moderate risk	Not applicable	Low risk	Low risk	Moderate risk	Low risk	Moderate risk
28.	van der Werf 2001	High risk	Not applicable	Low risk	Low risk	Moderate risk	Low risk	High risk

### Data analysis and synthesis

Data were analyzed using both qualitative (narrative) and quantitative syntheses. In the qualitative (narrative) synthesis, the characteristics, risk of bias, and methodological quality of the involved studies were summarized. The quantitative synthesis of the relationships between PSF and each of the outcomes of interest (mobility function, functional recovery, functional performance, and participation) involved a comprehensive meta-analysis of the correlation coefficient (r) values and sample sizes (N) of the included studies in the form of meta-correlation.

### Synthesis of the evidence profile

The Cochrane GRADE technique was applied to synthesize the evidence and rate the certainty of the evidence to arrive at a definite conclusion.

## Results

### Identification and selection of eligible studies

**Figure 1:**
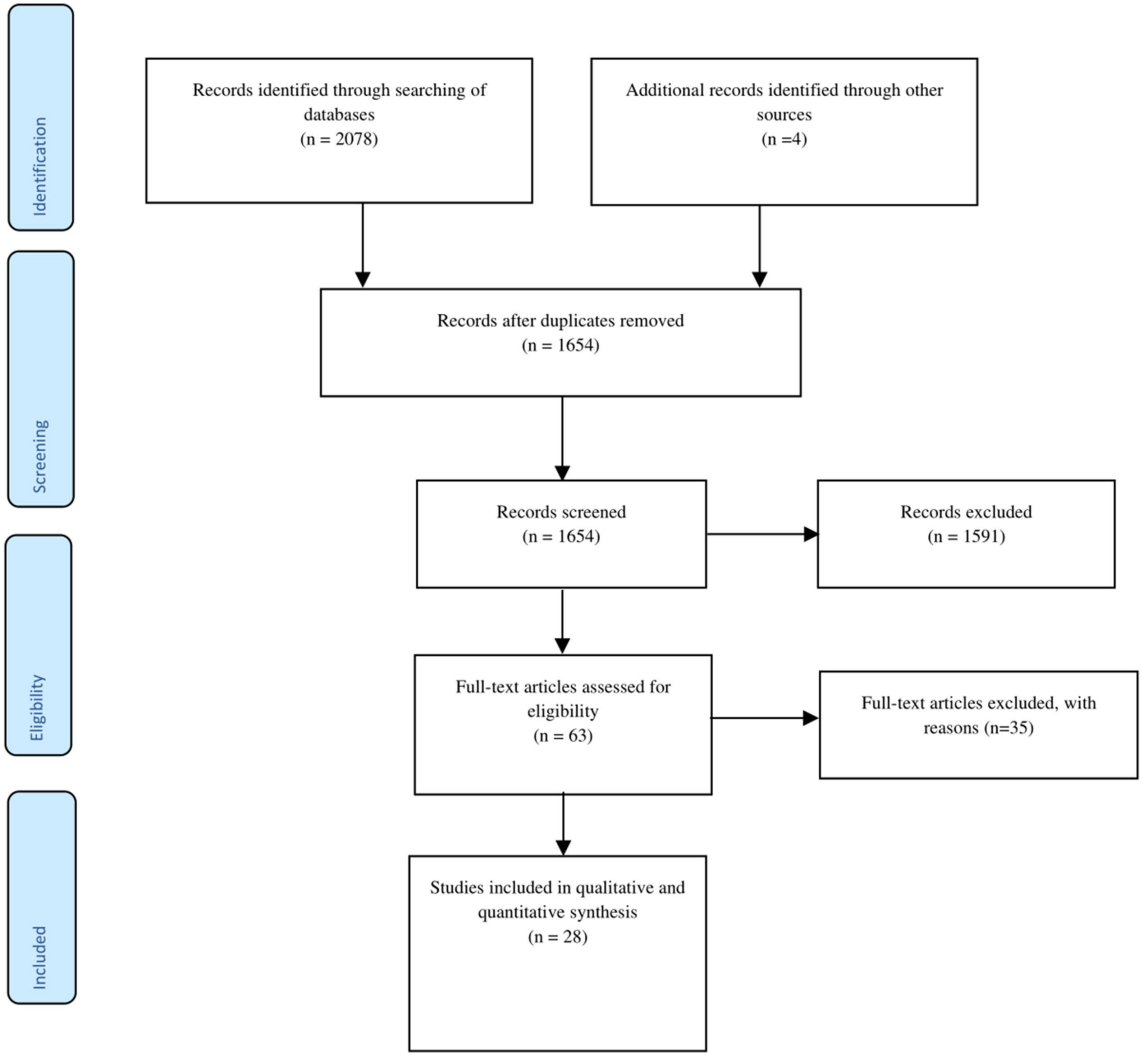
PRISMA flowchart of study selection. This figure illustrates the process of identification and selection of studies in the review.

The initial search of the online databases and other sources yielded a total of 2,082 relevant articles. Of these, 428 duplicates were removed, and the remaining 1,654 articles were subjected to title and abstract screening. Of these, 1,591 articles were excluded for failing to meet the eligibility requirements. The 63 remaining articles were then subjected to full-text screening against the inclusion and the exclusion criteria. Finally, 28 articles that met the eligibility criteria were included in the review. All of the studies were either cross-sectional (23 studies), longitudinal (3 studies), or observational (2 studies). Figure 1 presents the study identification and selection process in line with PRISMA guidelines.

### Description of the included studies

Twenty-eight studies are included in this review, and their detailed characteristics are presented in Table 1. Nine of these studies were conducted in the United States, eight in Europe (two in the Netherlands and one each in the United Kingdom, Switzerland, Norway, Sweden, Belgium, and Denmark), six in Asia (two in Hong Kong and one each in China, Turkey, India, and Korea), four in Africa (three in Nigeria and one in Egypt), and one in Australasia (one in Australia). A total of 2,495 stroke survivors participated in the included studies, of whom 1,626 and 869 participants were male and female, respectively. The age range of the participants was 26–89 years, with their mean age ± standard deviation across the studies ranging from 52.5 ± 9.5 to 71.1 ± 9.9 years.

The time since stroke among the participants ranged from 1 to 166 months, with the mean time since stroke/post-stroke duration across the studies ranging from 5 to 73.56 months. Twenty-three studies recruited chronic stroke survivors, five recruited sub-acute stroke survivors, and three recruited acute stroke survivors. Eleven studies reported stroke types and subtypes, 11 studies reported the affected hemisphere/side, and five studies reported the lesion/infarct location. Regarding the level of fatigue evaluated, 27 studies evaluated chronic fatigue, while three studies evaluated exertion/state fatigue. Sixteen studies used the Fatigue Severity Scale (FSS); six used the Fatigue Impact Scale (FIS) and modified FIS; two studies each used the Fatigue Assessment Scale (FAS), vitality component of the SF36, and the Visual Analogue Scale. One study each used the Numeric Rating Scale (NRS), the Fatigue Scale for Motor and Cognitive Functions (FSMC), the Fatigue Index Scale (FI), and the Checklist Individual Strength (CIS) Fatigue Scale. The outcomes evaluated in the studies included but were not limited to the following domains: lower extremity muscle strength, gait/walking, gait speed, walking endurance, walking/ambulatory activity, neuromuscular fatigability, walking economy, mobility, balance, motor performance, ADLs, physical functioning, activity, stroke severity/impairment, disability, QOL, participation, and self-efficacy/confidence.

### Reported measures of fatigue

Chronic and exertion/state fatigue were assessed in 24 and 4 studies, respectively. For the evaluation of chronic fatigue, the FSS was used in 16 studies, the FIS/modified FIS was used in six studies, the FAS was used in two studies, the vitality component of the SF36 (VITs) was used in two studies, and the FSMC, NRS, and CIS were each used in one study. For the evaluation of exertion/state fatigue, the Visual Analogue Scale was used in two studies, and the Fatigue Index (FI) Scale was used in one study.

### Reported outcomes

The included studies reported several outcomes. Six studies reported findings on mobility (1, 2, 12, 13, 25), six studies on gait speed (8, 19, 26–29), five studies on walking economy/aerobic capacity (3, 30–32), four studies on walking endurance (14, 26, 27, 32), three studies on walking/ambulatory activity (26, 33, 34), one study on cadence (29), and seven studies on ADLs (2, 12, 14, 15, 25, 35, 36). Furthermore, four studies reported findings on general motor performance (3, 19, 32, 35), five studies on lower extremity motor performance (2, 8, 12, 16, 19), six studies on upper extremity motor performance (2, 8, 12, 16, 17, 19), and one study on physical function (2). In addition, four studies reported findings on balance performance (8, 27, 29, 31), three studies on lower extremity functional muscle strength (8, 12, 16), two studies on knee extensor strength/neuromuscular fatigability (14, 37), and four studies on self-confidence/efficacy (27, 29, 31). Moreover, four studies reported findings on disability (38–41), two studies on activity and participation (12, 27), and three studies on QOL (1, 13, 36). Finally, two studies reported findings on stroke impairment/severity (36, 41).

### Reported outcome measures

Outcome measures such as the Bathel Index, the Modified Bathel Index, the 15-item ADL tool, the Lawton ADL scale, the Frenchay Activities Index, and the ACTIVLIM-Stroke Questionnaire were used across studies to evaluate ADLS. The Rivermead Mobility Index, Rivermead Motor Assessment (gross), Life Space Assessment, and 11-item mobility tool were employed among the studies to evaluate mobility function-related outcomes. In the studies, gait speed, walking endurance, and walking economy/aerobic fitness were assessed with a 10-m walk test, a 6-min walk test, and a graded exercise test, respectively. Five-Times-Sit-to-Stand test and dynamometers were used to evaluate lower limb functional muscle strength and knee extensor strength, respectively, in the studies. Across the studies, the Fugl-Meyer Assessment of Motor Recovery, Rivermead Motor Assessment, the Motricity Index, Lower Extremity Motor Function Tool, motor items of the Stroke Impairment Assessment Set, and Nine-Hole Peg test were employed to assess the motor function-related outcomes. Balance performance in the studies was evaluated with the Berg Balance Scale, Kinesthetic Ability Trainer, and Balance Evaluation Systems Test, while the Falls Efficacy Scale, Activity Specific Balance Confidence Scale, and Chronic Disease Self-Efficacy Scale were used to assess the self-efficacy/confidence in the studies. Stroke severity was examined using the National Institute of Health Stroke Scale. The Stroke Specific Quality of Life Questionnaire was used to evaluate the quality of life of stroke survivors in the studies. Among the studies, disability-related outcomes were evaluated with outcome measures such as the Modified Ranking Scale, Functional Independence Measure, and the Sickness Impact Scale. Participation-related outcomes were assessed using the Community Integration Measure and ICF Measure of Participation and Activities in the studies.

### Study quality assessment

Using the NIH 14-item quality assessment tool, all of the included studies were rated to have fair quality. The majority of the studies were deficient in elements related to items 5 (sample size justification), 6 (measuring of exposure prior to the outcome), 7 (sufficient time to see the effect of exposure on outcome), 10 (assessment of exposure more than once), 12 (blinding of outcome assessors), and 14 (confounders measurement and adjustment). Elements related to item 13 (follow-up assessment) were not applicable to most of the studies due to them being cross-sectional; similarly, elements in item 8 (exposure evaluation in relation to outcome) were not applicable to many of the studies because of the evaluation tools for the exposure used in the studies. All of the studies reported and scored items 1 (research question or objective), 2 (study population), 3 (participation rate), 4 (selection of subjects), 9 (exposure measures), and 11 (outcome measures), except one study that did not report item 3. Only four studies (12, 16, 19, 25) justified the sample sizes used, and most of the studies (19) had small sample sizes.

All except two studies evaluated the exposure and outcomes once and simultaneously, without having cause and effect. The two studies (33, 41) that evaluated more than once were longitudinal. Only one study (36) blinded the assessors, and only four studies (1, 8, 12, 37) adjusted for confounders. Almost all of the studies clearly defined the exposure and outcome measures for various outcomes. A total of 10 studies (13, 17, 25, 26, 28, 29, 33, 38, 39, 41) did not report the psychometric properties of the exposure or outcome measures. Nine studies (3, 13, 26–29, 32, 34, 39) did not specify the recruitment period of the participants, and eight studies (8, 13, 14, 26–28, 30, 32) did not specify the recruitment Centre. The inclusion criteria were not explicitly stated in two studies (37, 39), and the exclusion criteria were also not explicitly stated in two studies (30, 39). The study quality assessment and quality appraisal findings are presented in Table 2.

### ROB

With regard to ROB, of the 28 included studies, 16 studies were judged to have a low ROB (1, 2, 12, 15–17, 19, 25, 30, 31, 33, 36–38, 40, 41), nine had a moderate ROB (3, 13, 14, 27–29, 32, 34, 35), and three studies had a high ROB (8, 26, 39). In the domains of prognostic factor measurement, outcome measurement, and statistical analysis and reporting, all of the included studies had a low ROB, except one study that had a moderate ROB (8). In the domain of study confounding, all of the studies had a moderate ROB, except six studies that had a low ROB (1, 16, 30, 31, 37, 40) and one study that had a high ROB (8). The domain of study attrition was not applicable to most of the studies except three studies that had a low ROB in this domain (1, 33, 41) due to their longitudinal design. In the domain of study participation, most of the studies had a low or moderate ROB, except two studies that had a high ROB (26, 39). Details of the ROB assessment results are provided in Table 3.

### Analysis of the results

The main findings of the studies were categorized into four broad outcomes domains: mobility function-related outcomes (mobility, gait, walking economy/aerobic fitness, walking endurance, and walking/ambulatory activity), functional recovery-related outcomes (ADLs, motor performance, physical function, activity, stroke severity/impairments, and lower extremity functional muscle strength), functional performance-related outcomes (balance and self-confidence/efficacy), and participation-related outcomes (disability, participation, and QOL). We evaluated the relationship of PSF with each individual outcome within these four broad domains.

#### PSF and mobility function-related outcomes

##### Relationship between PSF and mobility function

**Figure 2:**
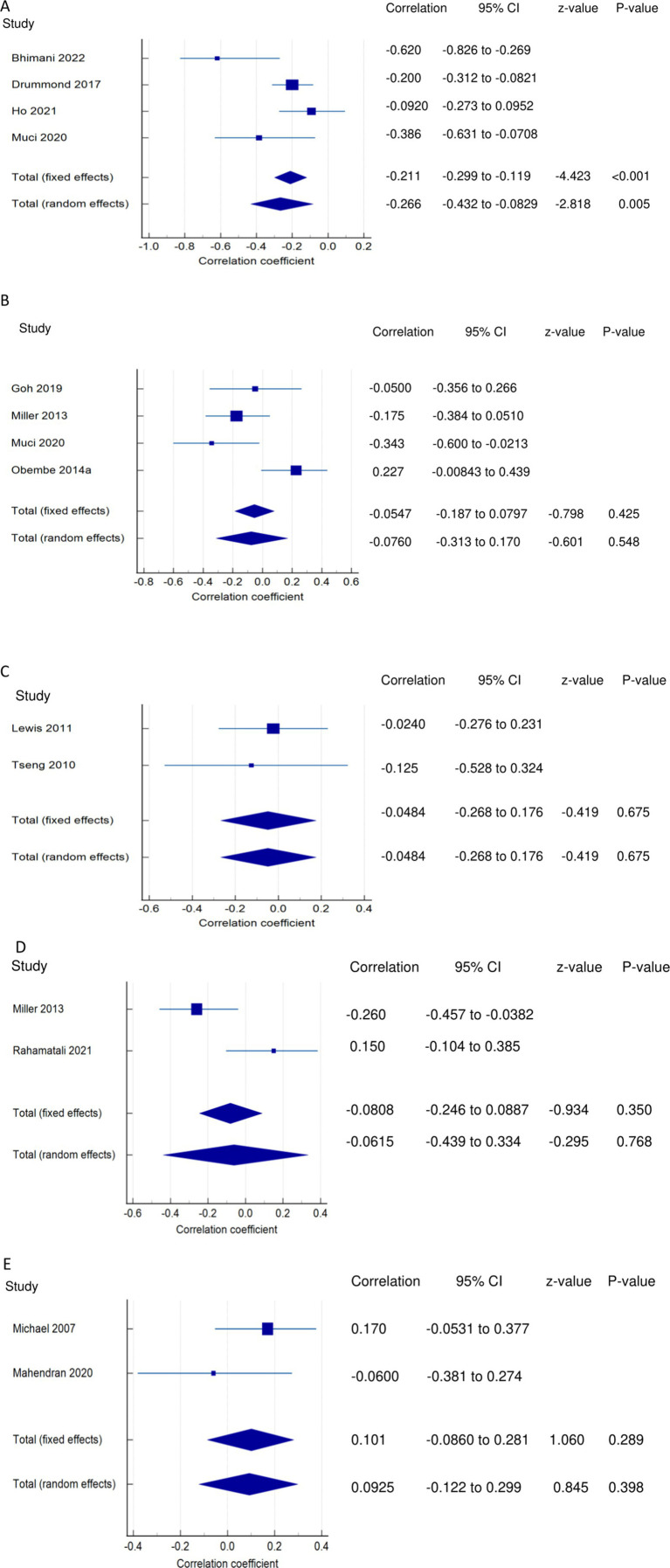
Relationship of PSF with mobility function related outcomes. This figure illustrates the relationship of PSF with **(A)** mobility; **(B)** gait speed; **(C)** walking economy; **(D)** walking endurance; **(E)** walking activity. The boxes represent point estimate and the size for each individual study, the horizontal lines represent the 95% CI and its boundaries for each individual study result, whereas the diamond shapes represent the pooled result estimate (summary of the meta-analysis) showing the overall relationship estimate for both fixed and random effects.

*PSF and mobility (narrative synthesis)*: Six studies reported a relationship between mobility and PSF, which was negative in all of these studies. This negative relationship was significantly strong in one study (2), significantly moderate in two studies (1, 13), significantly weak in two studies (19, 25), and non-significantly very weak in one study (12). PSF and mobility (quantitative synthesis): A statistically significant weak negative relationship (Figure 2A) was found between PSF and mobility in both fixed-effects (FE) (meta r = −0.211; *p* = <0.001) and random-effects (RE) meta-analyses (meta r = −0.266; *p* = 0.005), with statistically significant heterogeneity observed between the studies (Q = 7.8781; *p* = 0.0486; I^2^ = 61.92%; 95% CI for I^2^ = 0.00 to 87.22). Evidence profile synthesis: *Using the GRADE approach, as the included studies had a low ROB and fair methodological qualities, with a certain level of heterogeneity, the certainty of evidence was considered moderate for a weak negative correlation between PSF and mobility.*

##### Relationship between PSF and gait/walking function

*PSF and gait (narrative synthesis)*: Five studies showed a negative relationship between PSF and walking speed, which was significantly strong (26), weak (19), and non-significantly weak (29) each in one study and very weak in two studies (8, 27). In another study, gait speed was significantly related to cognitive fatigue but not to physical and overall fatigue (28). PSF and gait speed (quantitative synthesis):

Both FE and RE meta-analysis results revealed a statistically non-significant and very weak negative relationship (Figure 2B) between PSF and gait speed (*p* > 0.05), with statistically significant heterogeneity observed among the studies (Q = 9.6913; *p* = 0.0214; I^2^ = 69.04%). Evidence profile synthesis: *Using the GRADE approach, as half of the included studies had a low ROB and fair methodological qualities, with certain imprecision and substantial heterogeneity, the certainty of the evidence was considered low for the lack of a significant correlation between PSF and gait speed.* Additionally, the dynamic gait index had a significantly strong negative relationship with performance fatigability (26).

*PSF and walking economy/aerobic fitness (narrative synthesis)*: The studies revealed a negative relationship between PSF and walking economy/aerobic fitness. The relationship was not significant for chronic fatigue (3, 30, 31) but was significant and strong (32) and significant and moderate for exertion/state fatigue (3). PSF and walking economy/aerobic fitness (quantitative synthesis): Both FE and RE meta-analysis results showed a statistically non-significant and very weak negative relationship (Figure 2C) between PSF and walking economy (*p* > 0.05), and there was no significant heterogeneity among the studies (Q = 0.1414; *p* = 0.7069; I^2^ = 0.00%). Evidence profile synthesis: *Using the GRADE approach, as few of the included studies had a low or moderate ROB and fair methodological qualities, with no heterogeneity and certain imprecision, the certainty of evidence was considered low for the lack of significant correlation between PSF and walking economy.*

*PSF and walking endurance (narrative synthesis)*: Some of the included studies evaluated the relationship between walking endurance and chronic fatigue and/or exertion/acute fatigue. Chronic fatigue was reported to have a significant, weak negative relationship (27) and a non-significant, very weak positive relationship (14) with walking endurance. Exertion/state fatigue had non-significant, strong (32) and weak (26) negative relationships with walking endurance. PSF and walking endurance (quantitative synthesis): Both FE and RE meta-analysis results revealed a statistically non-significant and very weak negative relationship (Figure 2D) between PSF and walking endurance (*p* > 0.05), with statistically significant heterogeneity observed among the studies (Q = 5.7151; *p* = 0.0168; I^2^ = 82.50%). Evidence profile synthesis: *Using the GRADE approach, as few of the included studies had a moderate ROB and fair methodological qualities, with considerable heterogeneity and certain imprecision, the certainty of evidence was considered very low for the of a significant correlation between PSF and walking endurance.*

*PSF and walking/ambulatory activity (narrative synthesis)*: The studies showed opposing evidence on the relationship between PSF and walking/ambulatory activity. One study showed a non-significant, moderate (26) and very weak (33) negative relationship between PSF and walking/ambulatory activity, while another study (34) showed a non-significant, very weak positive relationship between PSF and walking/ambulatory activity. In another study, PSF had a significantly weak negative relationship with functional ambulation (8). PSF and walking activity (quantitative synthesis): Both the FE and RE meta-analysis results showed a statistically non-significant, very weak positive relationship (Figure 2E) between PSF and walking/ambulatory activity (*p* > 0.05), with no significant heterogeneity observed among the studies (Q = 1.2357; *p* = 0.2663; I^2^ = 19.07%). Evidence profile synthesis: *Using the GRADE approach, as few of the included studies had a low and moderate ROB and fair methodological qualities, with no heterogeneity and certain imprecision, the certainty of evidence was considered low for the lack of a significant correlation between PSF and walking activity.*

#### PSF and functional recovery-related outcomes

##### Relationships of PSF with ADLs

**Figure 3:**
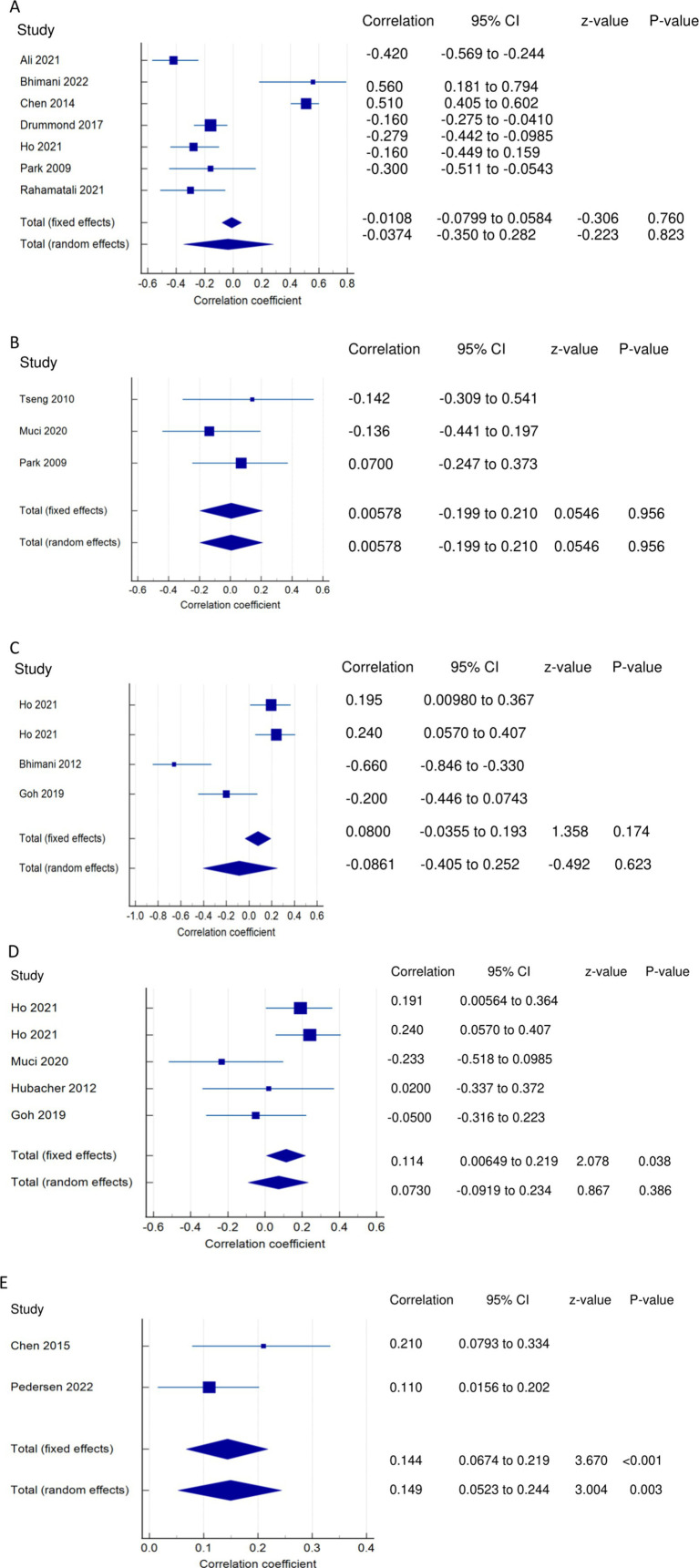
Relationship of PSF with functional recovery related outcomes. This figure illustrates the relationship of PSF with **(A)** ADLs; **(B)** general motor performance; **(C)** lower extremity motor performance; **(D)** upper extremity motor performance; **(E)** stroke impairment/severity. The boxes represent point estimate and the size for each individual study, the horizontal lines represent the 95% CI and its boundaries for each individual study result, whereas the diamond shapes represent the pooled result estimate (summary of the meta-analysis) showing the overall relationship estimate for both fixed and random effects.

*PSF and ADLs (narrative synthesis)*: Most studies revealed a negative relationship between PSF and ADL. The inverse relationship is significantly moderate (15), weak (12, 14), and very weak (25). On the other hand, the correlation was found to be non-significantly very weak negative (35) and significantly moderate positive (2, 36). PSF and ADLs (quantitative synthesis): Both the FE and RE meta-analysis results revealed a statistically non-significant and very weak negative relationship (Figure 3A) between PSF and ADLs (*p* > 0.05), with statistically significant heterogeneity observed among the studies (Q = 117.5112; *p* < 0.0001; I^2^ = 94.89%). Evidence profile synthesis: *Using the GRADE approach, as the majority of included studies had a low ROB and fair methodological qualities, with considerable heterogeneity and certain imprecision, the certainty of the evidence was considered very low for the lack of a significant correlation between PSF and ADLs.*

##### Relationships of PSF with motor performance

*PSF and general motor performance (narrative synthesis)*: Some studies reported a non-significant, very weak positive (3, 35) and negative (19) relationship between chronic PSF and motor performance. However, a significantly strong negative relationship was demonstrated between exertion/state fatigue and motor function (32). PSF and general motor performance (quantitative synthesis): Both the FE and RE meta-analysis results showed a statistically non-significant and very weak positive relationship (Figure 3B) between PSF and general motor performance (*p* > 0.05), with no significant heterogeneity observed among the studies (Q = 1.1836; *p* = 0.5533; I^2^ = 0.00%). Evidence profile synthesis: *Using the GRADE approach, as few included studies had a moderate ROB and fair methodological qualities, with no heterogeneity and certain imprecision, the certainty of the evidence was considered low for the lack of significant correlation between PSF and general motor performance.*

*PSF and lower extremity motor performance (LEMP) (narrative synthesis)*: Most of the studies showed a negative relationship between PSF and LEMP, with the correlations being significant and strong (2), non-significant and weak (8) or non-significant and very weak (19). One study reported a significant weak (16) and very weak (12) positive relationship between PSF and LEMP. PSF and lower extremity motor performance (LEMP) (quantitative synthesis): The FE and RE meta-analysis results revealed statistically non-significant, very weak positive and weak negative relationships, respectively (Figure 3C) between PSF and LEMP (*p* > 0.05), but there was statistically significant heterogeneity among the studies (Q = 22.9362; *p* = <0.0001; I^2^ = 86.92%). Evidence profile synthesis: *Using the GRADE approach, as the majority of included studies had a low ROB and fair methodological qualities, with considerable heterogeneity, the certainty of the evidence was considered low for the lack of a significant correlation between PSF and LEMP.*

*PSF and upper extremity motor performance (UEMP) (narrative synthesis)*: Three studies revealed a negative relationship between PSF and UEMP, with the correlations being significant and moderate (2), non-significant and weak (19), or non-significant and very weak (8). Three other studies showed a positive relationship between PSF and UEMP, with the relationships being significant and weak (16), significant and very weak (12), or non-significant and very weak (17). PSF and upper extremity motor performance (UEMP) (quantitative synthesis): A statistically significant, very weak positive relationship (Figure 3D) was found between PSF and UEMP in the FE meta-analysis (meta r = 0.114; *p* = 0.038) and RE meta-analysis (meta r = 0.0730; *p* = 0.386), with no significant heterogeneity among the studies (Q = 8.3411; *p* = 0.0799; I^2^ = 52.04%; 95% CI for I^2^ = 0.00 to 82.38). Evidence profile synthesis: *Using the GRADE approach, as the majority of the included studies had a low ROB and fair methodological qualities, with no significant heterogeneity and certain imprecision, the certainty of evidence was considered moderate for a significant, very weak positive correlation between PSF and UEMP.*

##### Relationships of PSF with stroke severity/impairment

*PSF and stroke severity/impairment (SI) (narrative synthesis):* One study each showed a significant weak positive (36) or very weak positive (41) relationship between PSF and stroke severity/impairment. PSF and stroke severity/impairment (SI) (quantitative synthesis): The meta-analysis results revealed statistically significant very weak positive (FE, meta r = 0.144; *p* < 0.001) and (RE, meta r = 0.149; *p* = 0.003) relationships (Figure 3E), between PSF and SI, with no significant heterogeneity among the studies (Q = 1.5090; *p* = 0.2193; I^2^ = 33.73%; 95% CI for I^2^ = 0.00 to 0.00). Evidence profile synthesis: *Using the GRADE approach, as few of the included studies had a low ROB and fair methodological qualities, with no heterogeneity, the certainty of the evidence was considered moderate for a significant, very weak positive correlation between PSF and stroke severity/impairment.*

##### Relationships of PSF with physical function and activity

*PSF and Physical function (PF) (narrative synthesis)*: PSF had a significantly strong negative relationship with PF (2).

*PSF and activity (narrative synthesis):* PSF had a significant, moderately positive relationship with activity (27).

##### Relationships of PSF with functional muscle strength

**Figure 4:**
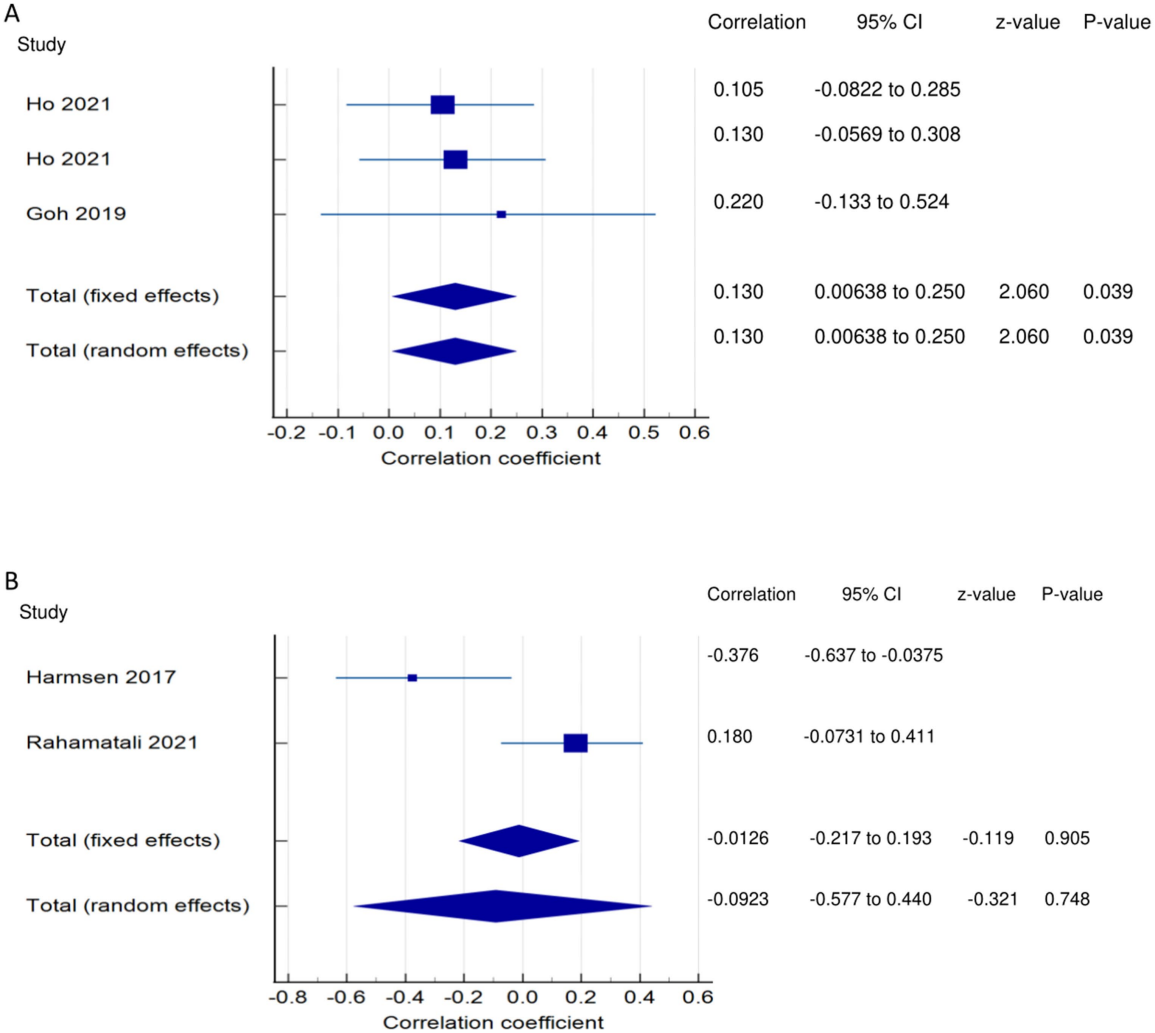
Relationship of PSF with functional recovery related outcomes. This figure illustrates the relationship of PSF with **(A)** lower extremity functional muscle strength; **(B)** knee extensor strength. The boxes represent point estimate and the size for each individual study, the horizontal lines represent the 95% CI and its boundaries for each individual study result, whereas the diamond shapes represent the pooled result estimate (summary of the meta-analysis) showing the overall relationship estimate for both fixed and random effects.

*PSF and lower extremity functional muscle strength (LEFMS) (narrative synthesis)*: One study and two studies reported non-significant weak (8) and very weak (12, 16) positive relationships, respectively, between PSF and LEFMS. Lower extremity functional muscle strength (LEFMS) (quantitative synthesis): Both the FE and RE meta-analysis results revealed a statistically significant, very weak positive relationship (Figure 4A) between PSF and LEFMS (meta r = 0.130; *p* = 0.039), with no significant heterogeneity among the studies (Q = 0.3291; *p* = 0.8483; I^2^ = 0.00%; 95% CI for I^2^ = 0.00 to 79.61). Evidence profile synthesis: *Using the GRADE approach, as the majority of the included studies had a low ROB and fair methodological qualities, with no heterogeneity, the certainty of evidence was considered moderate for a significant, very weak correlation between PSF and LEFMS.*

*PSF and knee extensor strength (KES)/neuromuscular fatigability (narrative synthesis):* Two studies showed opposing findings regarding the correlation between PSF and KES. One study showed a significantly weak negative relationship (37) and a non-significant, very weak positive relationship between PSF and KES (14). PSF and knee extensor strength/neuromuscular fatigability (KES) (quantitative synthesis): Both the FE and RE meta-analysis results revealed a statistically non-significant, very weak negative relationship (Figure 4B) between PSF and KES (*p* > 0.05), with statistically significant heterogeneity among the studies (Q = 6.6298; *p* < 0.0100; I^2^ = 84.92%). Evidence profile synthesis: *Using the GRADE approach, as few of the included studies had a low or moderate ROB and fair methodological qualities, with considerable heterogeneity and certain imprecision, the certainty of evidence was considered very low for the lack of significant correlation between PSF and KES.*

#### PSF and functional performance-related outcomes

**Figure 5:**
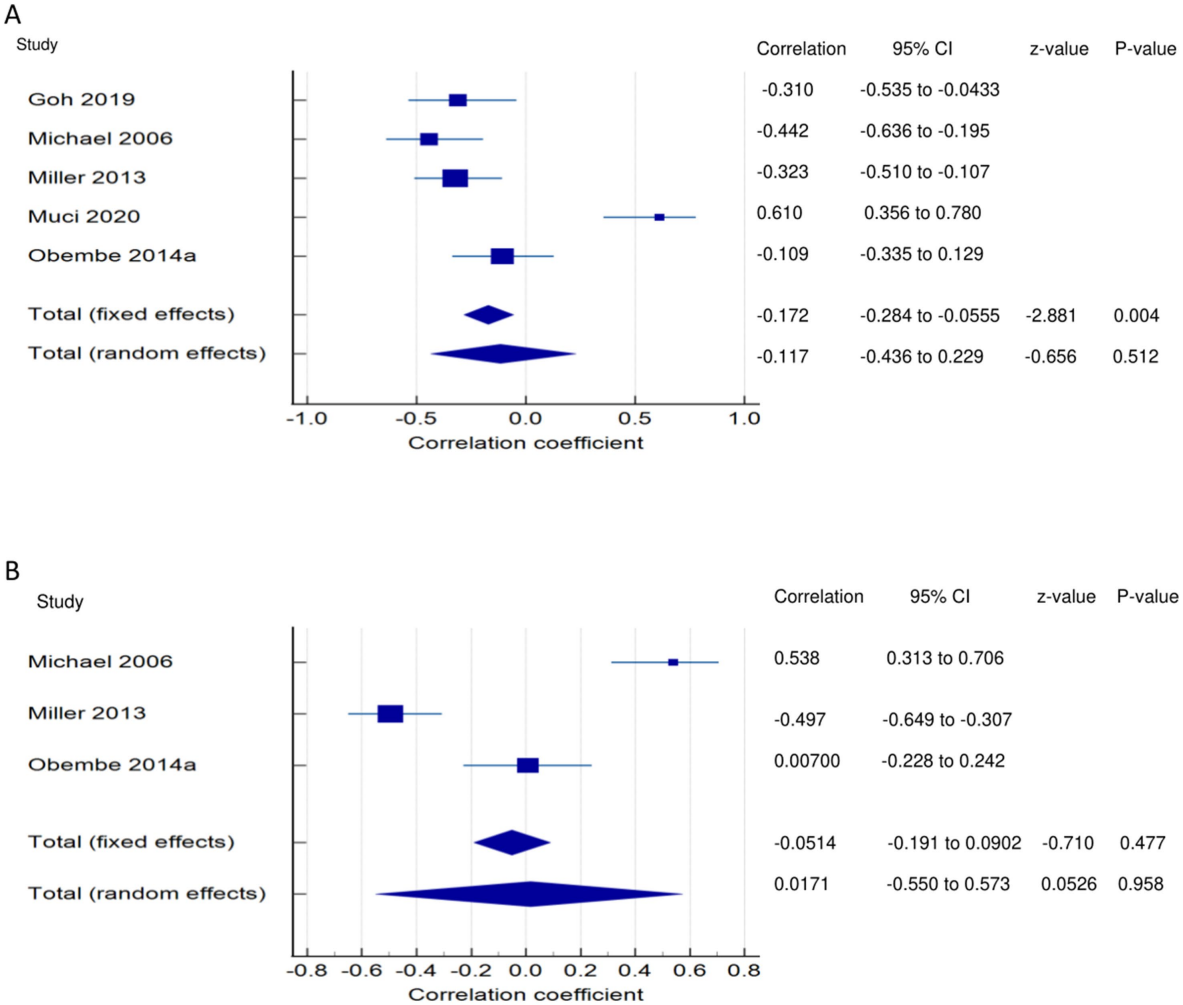
Relationship of PSF with functional performance related outcomes. This figure illustrates the relationship of PSF with **(A)** balance; **(B)** self-efficacy/confidence. The boxes represent point estimate and the size for each individual study, the horizontal lines represent the 95% Cl and its boundaries for each individual study result, whereas the diamond shapes represent the pooled result estimate (summary of the meta-analysis) showing the overall relationship estimate for both fixed and random effects.

*PSF and balance performance (narrative synthesis):* The majority of the studies showed a negative relationship between PSF and balance, with the correlations being significant and moderate (31), significant and weak (8, 27), or non-significant very weak (29). One study reported a significant correlation between PSF and balance performance (28), and another showed a significantly strong positive relationship with static balance (lower scores demonstrate better balance) (19). PSF and balance performance (quantitative synthesis): A statistically significant, very weak negative relationship (Figure 5A) was found between PSF and balance performance in the FE meta-analysis (meta r = −0.172; 95%; *p* = 0.004), with a non-significant, very weak negative relationship in the RE meta-analysis (*p* > 0.05). There was statistically significant heterogeneity among the studies (Q = 34.2972; *p* < 0.0001; I^2^ = 88.34%; 95% CI for I^2^ = 75.38 to 94.48). Evidence profile synthesis: *Using the GRADE approach, as half of the included studies had a low ROB and fair methodological qualities, with considerable heterogeneity and certain imprecision, the certainty of the evidence was considered low for a significant, very weak negative correlation between PSF and balance.*

*PSF and self-confidence/efficacy (narrative synthesis):* One study each showed a significant moderate negative (27) and positive (31) relationship between PSF and self-confidence/efficacy. However, in one study (29), the correlation was non-significant and very weakly positive. PSF and self-confidence/efficacy (quantitative synthesis): The FE and RE meta-analysis results revealed statistically non-significant, very weak negative and positive relationships, respectively, (Figure 5B) between PSF and self-confidence/efficacy (*p* > 0.05). There was statistically significant heterogeneity among the studies (Q = 39.5841; *p* < 0.0001; I^2^ = 94.95%). Evidence profile synthesis: *Using the GRADE approach, as most of the included studies had a low ROB and fair methodological qualities with considerable heterogeneity and certain imprecision, the certainty of the evidence was considered low for the lack of a correlation between PSF and self-efficacy.*

#### PSF and participation-related outcomes

**Figure 6:**
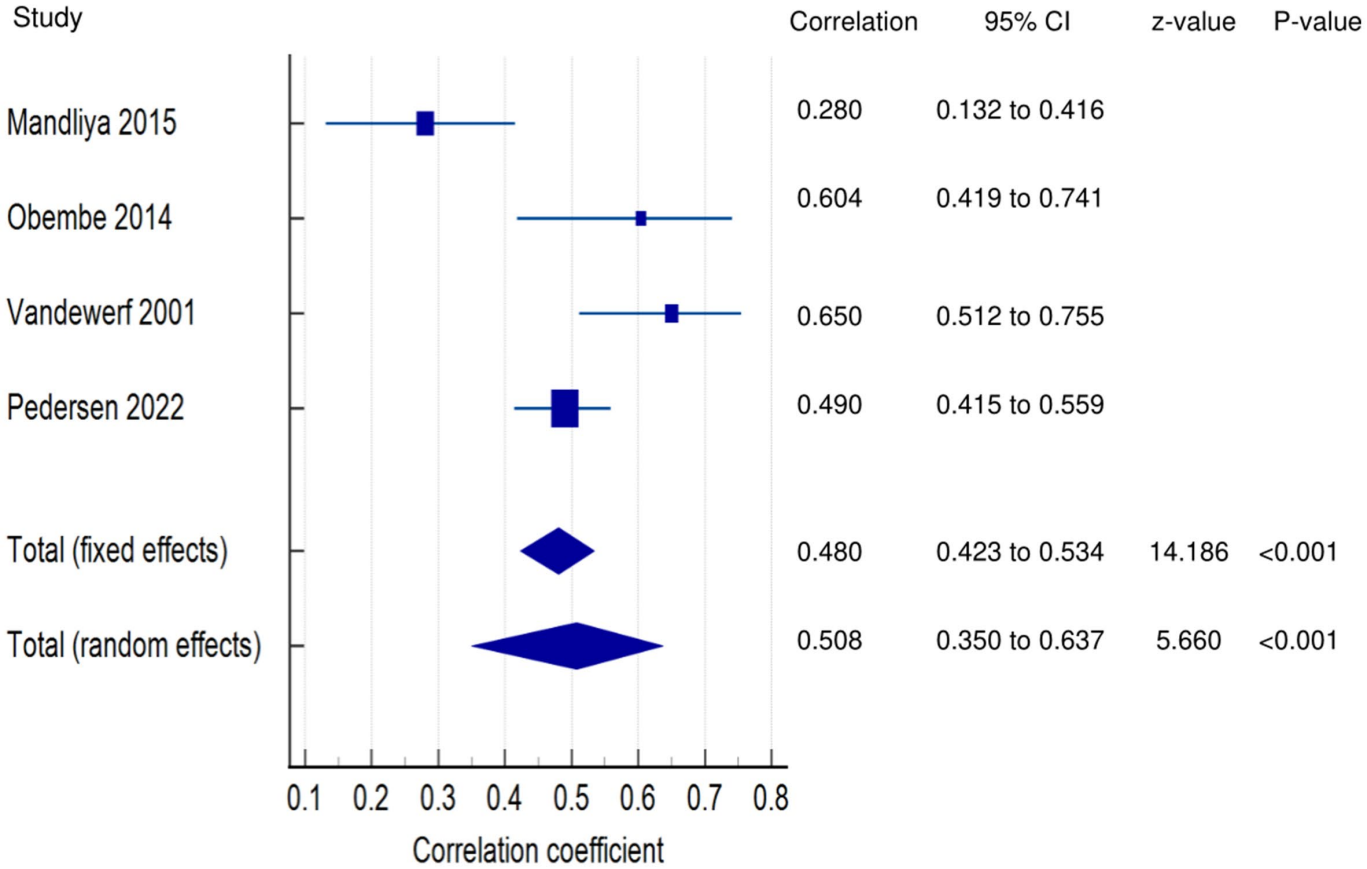
Relationship of PSF with participation related outcomes. This figure illustrates the relationship of PSF with disability. The boxes represent point estimate and the size for each individual study, the horizontal lines represent the 95% CI and its boundaries for each individual study result, whereas the diamond shapes represent the pooled result estimate (summary of the meta-analysis) showing the overall relationship estimate for both fixed and random effects.

*PSF and disability (narrative synthesis):* Two studies showed a significantly strong positive relationship (38, 39), and one each showed a moderate positive (41) and weak positive (40) relationship between PSF and disability. PSF and disability (quantitative synthesis): A statistically significant moderate positive relationship (Figure 6) was found between PSF and disability in the FE meta-analysis (meta r = 0.480; *p* < 0.001) and the RE meta-analysis (meta r = 0.508; *p* < 0.001), with statistically significant heterogeneity among the studies (Q = 16.3379; *p* = 0.0010; I^2^ = 81.64%; 95% CI for I^2^ = 52.31 to 92.93). Evidence profile synthesis: *Using the GRADE approach, as most of the included studies had a low ROB and fair methodological qualities, with considerable heterogeneity, the certainty of the evidence was considered moderate for a significant moderate positive correlation between PSF and disability.*

**Figure 7:**
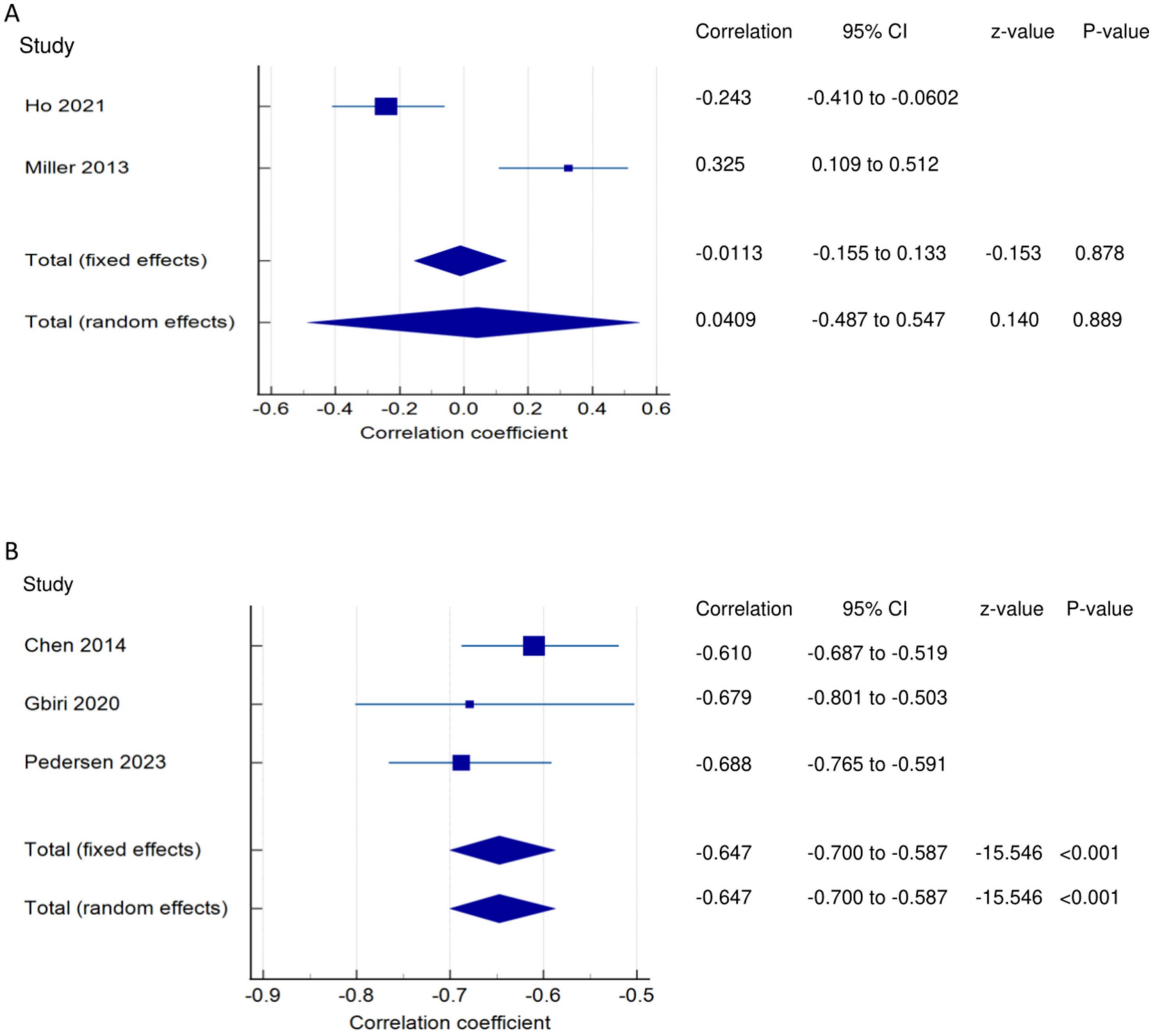
Relationship of PSF with participation related outcomes. This figure illustrates the relationship of PSF with **(A)** participation; **(B)** quality of life. The boxes represent point estimate and the size for each individual study, the horizontal lines represent the 95% CI and its boundaries for each individual study result, whereas the diamond shapes represent the pooled result estimate (summary of the meta-analysis) showing the overall relationship estimate for both fixed and random effects.

*PSF and participation (narrative synthesis):* In one study each, PSF was found to have a significant moderate positive relationship with activity (27), a significant weak relationship with participation (27), and a significant negative, weak relationship with community integration/participation (12). PSF and Participation (quantitative synthesis): Both the FE and RE meta-analysis results showed statistically non-significant, very weak negative and positive relationships, respectively (Figure 7A) between PSF and participation (*p* > 0.05), with statistically significant heterogeneity among the studies (Q = 15.0937; *p* = 0.0001; I^2^ = 93.37%). Evidence profile synthesis: *Using the GRADE approach, as few of the included studies had a low or moderate ROB and fair methodological qualities, with considerable heterogeneity, the certainty of the evidence was considered low for the lack of a correlation between PSF and participation.*

*PSF and QOL (narrative synthesis):* Three studies showed a significantly strong negative relationship between PSF and QOL (1, 13, 36). PSF and QOL (quantitative synthesis): Both FE and RE meta-analysis results revealed a statistically and significantly strong negative relationship (Figure 7B) between PSF and QOL (meta r = −0.647; *p* < 0.001), with no significant heterogeneity among the studies (Q = 1.7443; *p* = 0.4180; I^2^ = 0.00%; 95% CI for I^2^ = 0.00 to 96.15). Evidence profile synthesis: *Using the GRADE approach, as most of the included studies had a low ROB and fair methodological qualities with no heterogeneity, the certainty of the evidence was considered moderate for a significantly strong negative correlation between PSF and QOL.*

### Exertion/acute fatigue

#### Relationship between exertion/acute fatigue and walking economy

**Figure 8:**
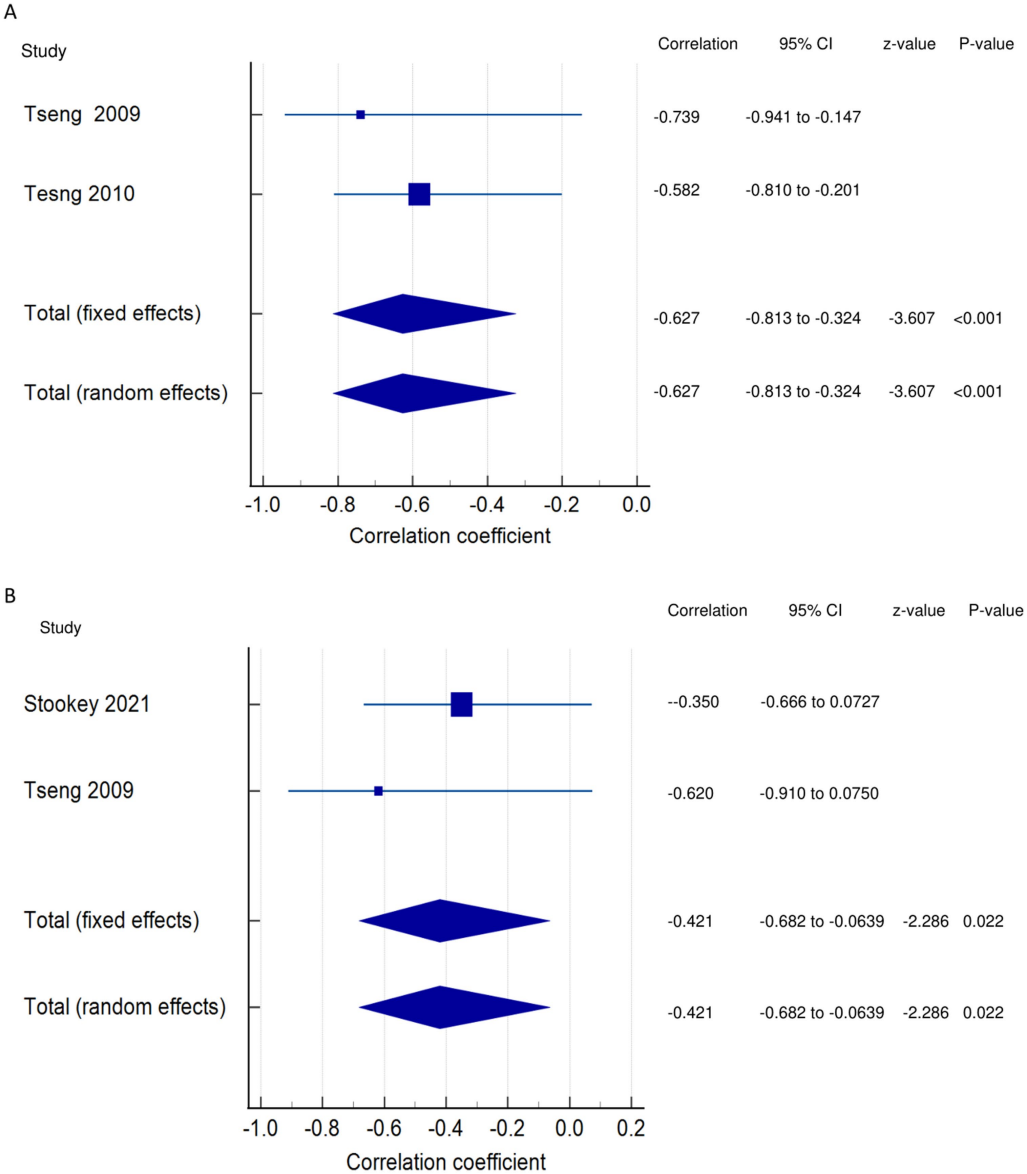
Relationship of exertion/acute fatigue with walking. This figure illustrates the relationship of exertion/acute fatigue with **(A)** walking economy; **(B)** walking endurance. The boxes represent point estimate and the size for each individual study, the horizontal lines represent the 95% CI and its boundaries for each individual study result, whereas the diamond shapes represent the pooled result estimate (summary of the meta-analysis) showing the overall relationship estimate for both fixed and random effects.

*Walking economy and PSF (exertion/acute fatigue):* Both the FE and RE meta-analysis results of both FE and RE revealed a statistically and significantly strong negative relationship (Figure 8A) between PSF (exertion/acute) and walking economy (meta r = −0.627; *p* < 0.001), with no significant heterogeneity among the studies (Q = 0.3599; *p* = 0.5486; I^2^ = 0.00%; 95% CI for I^2^ = 0.00 to 0.00). Evidence profile synthesis: *Using the GRADE approach, as few of the included studies had a moderate ROB and fair methodological qualities, with no heterogeneity and certain imprecision, the certainty of the evidence was considered low for a significantly strong negative correlation between PSF (exertion) and walking economy.*

#### Relationship between exertion/acute fatigue and walking endurance

*Walking endurance and PSF (exertion/acute fatigue)*: Both the FE and RE meta-analysis results showed a statistically significant moderate negative relationship (Figure 8B) between PSF (exertion/acute) and walking endurance (meta r = −0.421; *p* = 0.022), with no significant heterogeneity among the studies (Q = 0.5967; *p* = 0.4398; I^2^ = 0.00%; 95% CI for I^2^ = 0.00 to 0.00). Evidence profile synthesis: *Using the GRADE approach, as few of the included studies had a moderate or high ROB and fair methodological qualities, with no heterogeneity and certain imprecision, the certainty of evidence was considered low for a significant moderate negative correlation between PSF (exertion) and walking endurance.*

## Discussion

To the best of our knowledge, this is the first systematic review and meta-analysis to establish the scientific evidence on the relationships of PSF with mobility, recovery, performance, and participation of stroke survivors. Studies have shown that components of mobility-related function, including mobility, gait speed, walking economy, and functional ambulation, have significant negative relationships with PSF, with correlation strengths ranging from strong to moderate to weak. However, walking activity has no significant correlation with PSF. In the meta-analysis, only mobility showed statistically significant weak negative relationships with PSF, with moderate to low certainty of evidence. This suggests that greater PSF is associated with less mobility. These findings are supported by a previous study that reported mobility deficit severity to be related to fatigue, with a consequent reduction in home and community ambulatory activity in stroke survivors (31). The weak correlations obtained with some variables in our meta-analysis may be due to a lack of control of some confounding variables in some of the included studies or to the small number of studies evaluating those variables. Thus, future correlation studies should adjust for confounding factors to verify the existing correlations, and RCTs to involve training in relation to PSF are warranted. It is vital to differentiate between walking economy/aerobic fitness, walking endurance, and walking activity. Aerobic fitness has to do with submaximal and peak VO_2_ during walking exercise testing (3, 30), walking endurance has to do with distance covered while walking during a 6-min walk test (27), whereas walking activity involves total stride count or number of steps during walking (26, 31, 33, 34).

Our results revealed that components of functional recovery, such as ADLs, motor performance, physical function, and KES, had significant negative relationships with PSF. However, stroke severity/impairments were positively correlated with PSF, and lower extremity functional muscle strength showed no correlation with PSF. The strengths of the correlations ranged from strong and moderate to weak and very weak. The meta-analysis showed that stroke impairment had significant, very weak positive correlations with PSF, with moderate certainty of evidence. This suggests that greater stroke impairment is associated with greater PSF. This finding is supported by other findings that functional limitations because of fatigue commonly occur in stroke survivors (38) and that stroke survivors, in turn, tend to attribute more functional limitations to their fatigue (42).

Some of the included studies revealed contradictory relationships, in terms of the direction (negative, positive) and significance (significant, non-significant), between some recovery/function variables and PSF. Such discrepancies may be attributable to the lack of control over factors influencing the study outcomes, the use of different outcome measures, and the nature of the recruited stroke population, among others. This indicates the need for further studies to explore the relationship between PSF and such variables while controlling for confounding factors.

Some studies also reported that components of functional performance, such as balance performance and self-confidence, had a significant negative relationship with PSF. The meta-analysis findings revealed that balance performance had significant, very weak negative correlations with PSF, with low certainty of evidence. This suggests that greater PSF is associated with reduced balance performance and self-efficacy/confidence. This result is backed by the finding that the severity of fatigue in stroke survivors is significantly associated with Berg balance scale scores and self-efficacy (31). Although the direction of the correlation is majorly negative and the strength of the correlation ranges from moderate to weak, a few studies reported contradictory findings of positive correlations between PSF and balance performance and self-efficacy/confidence. This discrepancy may be attributable to the study settings, outcome measures used, and methodological limitations, especially those related to confounder adjustments and study selection. Thus, further studies are warranted to confirm our results.

Studies on disability have reported a significant positive relationship (strong, moderate, and weak) between disability and PSF. In the meta-analysis, disability showed a significant moderate positive correlation with PSF, with a moderate certainty of evidence. This suggests that a greater PSF is associated with greater post-stroke disability. This is expected, as PSF has been reported to be substantially related to post-stroke disability (39, 40). The findings of the studies are uniform in terms of the correlation direction, which supports the relationship. Hence, future RCTs aimed at reducing disability and its impact on PSF in stroke survivors are warranted.

The included studies demonstrated a significant negative relationship (strong and weak) between PSF and participation and QOL. In the meta-analysis, PSF was found to have a non-significant, very weak negative correlation with participation (low certainty of the evidence) and a significantly strong negative correlation with QOL (moderate certainty of the evidence). In the studies, the correlation direction was reported to be negative. These findings suggest that greater PSF is associated with reduced post-stroke participation and QOL. This result is supported by a previous finding that fatigue adversely affects QOL, rehabilitation outcomes, social participation, functional independence, and return to work among stroke survivors (43). Longitudinal studies are recommended to conduct follow-ups and determine how PSF influences post-stroke participation and QOL.

The included studies demonstrated significantly strong negative correlations of exertion/acute fatigue with walking activity and general motor performance. In the meta-analysis, exertion/acute fatigue showed a significantly strong negative correlation (low certainty of evidence) with walking economy and a significant moderate negative correlation (low certainty of evidence) with walking endurance. This suggests that greater exertion/acute PSF is associated with reduced post-stroke mobility. This is supported by a previous finding that mobility deficit is strongly associated with post-stroke fatigability, with compromised mobility and functional limitations increasing the incidence of fatigue (26). We found that few studies have reported on the impact of exertion/acute fatigue; hence, further studies are warranted to explore the effects of exertion/acute fatigue on the outcomes of interest in stroke survivors. Additionally, few studies have reported correlations with the components of fatigue; hence, future studies are required to assess the relationships of components of fatigue, such as physical and mental/cognitive fatigue, with the outcomes of interest. The review findings are crucial, and the implication is that since PSF has been found to be associated with reduced mobility, performance, and participation and increased impairment and disability in stroke, it is, therefore, crucial to have in place interventions and strategies targeted at reducing PSF in stroke survivors. Although fatigue differs from sleep, numerous subjective measures of sleep were found to correlate with fatigue, with sleep quality contributing to fatigue in chronic stroke survivors a year or more post-stroke (12). Additionally, this sleep quality was found to correlate positively with fatigue and independently predict it post-stroke, with poor sleep quality likely resulting in post-stroke daytime sleepiness (12). However, sleep quality was reported to likely be a target element in interventions for improving fatigue (12). Thus, it may be possible that interventions aimed at enhancing sleep quality post-stroke may also affect PSF.

### Strengths and limitations

The main strengths of this review are the literature search conducted in important databases for articles, the use of recommended tools for the assessment of methodological quality and ROB, adherence to PRISMA guidelines for conducting and reporting the review, and the use of Cochrane GRADE criteria for evidence synthesis. All the tools and guidelines/criteria facilitated clear evidence synthesis and the drawing of meaningful conclusions.

The review has some limitations. Only articles published in English were used, which may have resulted in selection bias. Many of the included studies were cross-sectional in nature. Thus, we could not establish a cause-and-effect relationship among the variables. Furthermore, the low certainty of evidence obtained for some correlations possibly signifies that the relationships may go in either positive or negative directions. Hence, some of the findings should be interpreted with caution, considering these limitations.

The main limitations of the included studies should also be taken into account. Some of the studies had small samples, which may limit the generalization of the findings, and many studies did not control or adjust for confounders, which may have affected their outcomes. Furthermore, the use of self-reported measures for fatigue in most of the studies might have affected the reliability of the evaluations. Additionally, some studies had limitations in sample selection and study participation. The majority of the studies did not categorize or report specific correlations with fatigue components such as physical and mental/cognitive components.

## Conclusion

This review and meta-analysis found that chronic PSF had significant negative correlations with mobility (moderate certainty of evidence), balance performance (low certainty of evidence), and quality of life (moderate certainty of evidence). It was also found to have a significant negative relationship with functional motor recovery in the narrative synthesis, though this was not supported by the meta-analysis. Additionally, chronic PSF revealed significant positive correlations with stroke severity/impairment (moderate certainty of evidence) and disability (moderate certainty of evidence).

For exertion/acute fatigue, our results revealed significant negative correlations of PSF with walking economy and walking endurance, both with low certainty of evidence. The narrative synthesis identified revealed significant negative correlations between exertion/acute fatigue and walking activity, as well as general motor performance.

## Data Availability

The data analyzed in this study is subject to the following licenses/restrictions: Data will be provided on reasonable request. Requests to access these datasets should be directed to shamay.ng@polyu.edu.hk.
