# Supplementary material for: Relationships of post-stroke fatigue with mobility, recovery, performance, and participation-related outcomes: a systematic review and meta-analysis
Source: Front Neurol. 2024 Oct 8;15:1420443. doi: 10.3389/fneur.2024.1420443 (PMC11493601; doi:10.3389/fneur.2024.1420443)
Supplement: Supplementary file 1 [file Data_Sheet_1.PDF]

Supplementary Material 1: Search strategy used in PubMed online data base and similar strategy was used in other data bases

("Stroke"[MeSH Terms] OR "Hemorrhagic Stroke"[MeSH Terms] OR "Ischemic Stroke"[MeSH Terms] OR "Brain Infarction"[MeSH Terms] OR "Hemiplegia"[MeSH Terms] OR "Cerebrovascular Disorders"[MeSH Terms] OR "Paresis"[MeSH Terms] OR ("Stroke"[MeSH Terms] OR "Stroke"[All Fields] OR "strokes"[All Fields] OR "stroke s"[All Fields] OR "Hemorrhagic Stroke"[All Fields] OR "Ischemic Stroke"[All Fields] OR "Brain Infarction"[All Fields] OR ("Hemiplegia"[MeSH Terms] OR "Hemiplegia"[All Fields] OR "hemiplegias"[All Fields]) OR "Cerebrovascular Disorders"[All Fields] OR ("Paresis"[MeSH Terms] OR "Paresis"[All Fields] OR "pareses"[All Fields]) OR ("cebrovascular"[All Fields] AND ("accidence"[All Fields] OR "accident s"[All Fields] OR "accidents"[MeSH Terms] OR "accidents"[All Fields] OR "accident"[All Fields])) OR "Post stroke"[All Fields])) AND ("Fatigue"[MeSH Terms] OR "fatigue syndrome, chronic"[MeSH Terms] OR "Mental Fatigue"[MeSH Terms] OR ("fatiguability"[All Fields] OR "fatiguable"[All Fields] OR "Fatigue"[MeSH Terms] OR "Fatigue"[All Fields] OR "fatigued"[All Fields] OR "fatigues"[All Fields] OR "fatiguing"[All Fields] OR "fatigueability"[All Fields] OR "post stroke fatigue"[All Fields] OR "physical fatigue"[All Fields] OR "Mental Fatigue"[All Fields] OR "cognitive fatigue"[All Fields] OR "fatigue syndrome chronic"[All Fields])) AND ("Postural Balance"[MeSH Terms] OR "Recovery of Function"[MeSH Terms] OR "Gait"[MeSH Terms] OR "gait disorders, neurologic"[MeSH Terms] OR "Walking Speed"[MeSH Terms] OR "Walking"[MeSH Terms] OR "Mobility Limitation"[MeSH Terms] OR "Locomotion"[MeSH Terms] OR "Lower Extremity"[MeSH Terms] OR ("Functional recovery"[All Fields] OR ("balance"[All Fields] OR "balanced"[All Fields] OR "balances"[All Fields] OR "balancing"[All Fields]) OR "static balance"[All Fields] OR "dynamic balance"[All Fields] OR "single-task balance"[All Fields] OR "dual-task balance"[All Fields] OR "postural sway"[All Fields] OR ("Gait"[MeSH Terms] OR "Gait"[All Fields]) OR "gait outcomes"[All Fields] OR ("walked"[All Fields] OR "Walking"[MeSH Terms] OR "Walking"[All Fields] OR "walks"[All Fields]) OR "gait spatiotemporal parameters"[All Fields] OR "gait kinematics"[All Fields] OR "gait kinetics"[All Fields] OR "gait disorders"[All Fields] OR ("ambulant"[All Fields] OR "ambulate"[All Fields] OR "ambulated"[All Fields] OR "ambulates"[All Fields] OR "ambulating"[All Fields] OR "ambulations"[All Fields] OR "ambulator"[All Fields] OR "ambulators"[All Fields] OR "Walking"[MeSH Terms] OR "Walking"[All Fields] OR "ambulation"[All Fields]) OR "motor function"[All Fields] OR "motor recovery"[All Fields] OR "motor impairment"[All Fields] OR "Lower Extremity"[All Fields] OR "gait pattern*"[All Fields

Supplementary Material 2: Adapted Quality in Prognosis Studies (QUIPS) evaluation tool and method of evaluation

The modified version of the QUIPS evaluation tool was adapted from the following sources:

Hayden JA, van der Windt DA, Cartwright JL, Côté P, Bombardier C. Assessing bias in studies of prognostic factors. Annals of internal medicine. 2013 Feb 19;158(4):280-6.

Hayden JA, Côté P, Bombardier C. Evaluation of the quality of prognosis studies in systematic reviews. Annals of internal medicine. 2006 Mar 21;144(6):427-37.

1. Study Participation

Prompting items (adapted): This domain evaluates if the study sample represents the population of interest (stroke survivors) sufficiently including adequate participation by eligible individuals, adequate description of source population for key characteristics, sufficient description of baseline study sample for key characteristics (such as some basic sociodemographic and clinical variables), as well as adequate description of: sampling frame and recruitment, time and location of recruitment, and inclusion and exclusion criteria. This domain entails rating the risk of bias (ROB) in selection.

ROB appraisal: The study participation domain was evaluated to be of low, moderate or high ROB for each study based on how satisfactory or not are the above prompting items. The evaluation was also based on the likelihood or not of differences in relationship of post stroke fatigue with mobility, recovery, performance, or participation related outcomes for participants and eligible nonparticipants.

Ratings:

- **Low ROB**: The relationship of post stroke fatigue with mobility, recovery, performance, or participation related outcomes is unlikely to be different for participants and eligible nonparticipants.
- **Moderate ROB**: The relationship of post stroke fatigue with mobility, recovery, performance, or participation related outcomes may be different for participants and eligible nonparticipants.
- **High ROB**: The relationship of post stroke fatigue with mobility, recovery, performance, or participation related outcomes is very likely to be different for participants and eligible nonparticipants.

2. Study Attrition

Prompting items (adapted): This domain evaluates the risk of attrition (drop out). This involves availability of study data (participants not lost to follow up sufficiently represent the study sample). The items include: adequate response rate for study participants, description of attempts to collect information on participants who dropped out, provision of reasons for loss to follow-up, adequate description of participants lost to follow-up, there are no vital differences between participants who completed the study and those who did not. The attrition was only evaluated in relevant studies; thus, it was not evaluated in cross sectional studies in the review. This domain entails rating the ROB in study attrition.

ROB appraisal: The study attrition domain was evaluated to be of low, moderate or high ROB for each study based on how satisfactory or not are the prompting items above which focus on attrition/drop out, and available participants for analysis. The evaluation was also based on the likelihood or not of differences in relationship of post stroke fatigue with mobility, recovery, performance, or participation related outcomes for completing and non-completing participants.

Ratings:

- **Low ROB**: The relationship of post stroke fatigue with mobility, recovery, performance, or participation related outcomes is unlikely to be different for completing and non-completing participants

Moderate ROB: The relationship of post stroke fatigue with mobility, recovery, performance, or participation related outcomes may be different for completing and non-completing participants

High ROB: The relationship of post stroke fatigue with mobility, recovery, performance, or participation related outcomes is very likely to be different for completing and non-completing participants

3. Prognostic Factor Measurement

Prompting items (adapted): This domain evaluates the measurement of post stroke fatigue for all participants in a similar way. The items include: a clear definition or description of the post stroke fatigue is provided, sufficiently valid and reliable method of post stroke fatigue measurement, continuous variables are reported or suitable (that is, not data-dependent) cut points are used, the method and setting of measurement of post stroke fatigue is the same for all study participants, adequate proportion of the study sample has complete data for the post stroke fatigue, appropriate methods of imputation are used for missing post stroke fatigue data. This domain entails rating the ROB associated with post stroke fatigue measurement.

ROB appraisal: The prognostic factor measurement domain was evaluated to be of low, moderate or high ROB for each study based on how satisfactory or not are the prompting items above which focus on post stroke fatigue. The evaluation was also based on the likelihood or not of differences in measurement of post stroke fatigue for different levels of mobility, recovery, performance, or participation related outcomes.

Ratings:

Low ROB: The measurement of post stroke fatigue is unlikely to be different for different levels of mobility, recovery, performance, or participation related outcomes.

Moderate ROB: The measurement of post stroke fatigue may be different for different levels of mobility, recovery, performance, or participation related outcomes.

High ROB: The measurement of post stroke fatigue is very likely to be different for different levels of mobility, recovery, performance, or participation related outcomes.

4. Outcome Measurement

Prompting items (adapted): This domain evaluates the measurement of mobility, recovery, performance, or participation related outcomes for all participants in a similar way. The items include providing a clear definition of mobility, recovery, performance, or participation related outcomes, adequately valid and reliable outcome measures and method of measurement of mobility, recovery, performance, or participation related outcomes, the method and setting of measurement of mobility, recovery, performance, or participation related outcomes is the same for all study participants.

Risk of bias appraisal: The outcome measurement domain was evaluated to be of low, moderate or high ROB for each study based on how satisfactory or not are the prompting items above which focus on the measurement of mobility, recovery, performance, or participation related outcomes. The evaluation was also based on likelihood or not of differences in measurement of mobility, recovery, performance, or participation related outcomes related to the baseline level of post stroke fatigue. This domain entails rating the ROB associated with outcomes of interest measurement.

Ratings:

Low ROB: The measurement of mobility, recovery, performance, or participation related outcomes is unlikely to be different related to the baseline level of post stroke fatigue.

Moderate ROB: The measurement of mobility, recovery, performance, or participation related outcomes may be different related to the baseline level of post stroke fatigue.

High ROB: The measurement of mobility, recovery, performance, or participation related outcomes is very likely to be different related to the baseline level of post stroke fatigue.

5. **Study Confounding**

Prompting items (adapted): This domain evaluates appropriate accounting of vital potential confounding factors. The items include: all important confounders are measured, clear definitions of the important confounders measured are provided, adequately valid and reliable measurement of all important confounders, the method and setting of confounding measurement are the same for all study participants, appropriate methods are used if imputation is used for missing confounder data, important potential confounders are accounted for in the study design, important potential confounders are accounted for in the analysis. This domain entails rating the ROB in study confounding.

Risk of bias appraisal: The study confounding domain was evaluated to be of low, moderate or high ROB for each study based on how satisfactory or not are the prompting items above which focus on the confounders. The evaluation was also based on likelihood or not of distortion in the observed relationship of post stroke fatigue with mobility, recovery, performance, or participation related outcomes by another factor related to post stroke fatigue and mobility, recovery, performance, or participation related outcomes.

Ratings:

Low ROB: The observed relationship of the post stroke fatigue with mobility, recovery, performance, or participation related outcomes is unlikely to be distorted by another factor related to post stroke fatigue and mobility, recovery, performance, or participation related outcomes.

Moderate ROB: The observed relationship of the post stroke fatigue with mobility, recovery, performance, or participation related outcomes may be distorted by another factor related to post stroke fatigue and mobility, recovery, performance, or participation related outcomes.

High ROB: The observed relationship of the post stroke fatigue with mobility, recovery, performance, or participation related outcomes is very likely to be distorted by another factor related to post stroke fatigue and mobility, recovery, performance, or participation related outcomes.

6. **Statistical Analysis and Reporting**

Prompting items (adapted): This domain evaluates the appropriateness of statistical analysis, and all primary outcomes of interest reported. The items include: sufficient presentation of data to assess the adequacy of the analytic strategy, strategy for model building is appropriate and is based on a conceptual framework or model, the selected statistical model is adequate for the design of the study, there is no selective reporting of results. This domain entails rating the ROB associated with statistical analysis and reporting.

Risk of bias appraisal: The statistical analysis and reporting domain was evaluated to be of low, moderate or high ROB for each study based on how satisfactory or not are the prompting items above which focus on the statistical analysis and reporting. The evaluation was also based on the likelihood or not of being spurious or biased in the reported results related to analysis or reporting.

Ratings:

Low ROB: The reported results are unlikely to be spurious or biased related to analysis or Reporting.

Moderate ROB: The reported results may be spurious or biased related to analysis or reporting.

High ROB: The reported results are very likely to be spurious or biased related to analysis or reporting.
